# The Clinical and Mutational Spectrum of Bardet–Biedl Syndrome in Saudi Arabia

**DOI:** 10.3390/genes15060762

**Published:** 2024-06-11

**Authors:** Doaa Milibari, Sawsan R. Nowilaty, Rola Ba-Abbad

**Affiliations:** 1 Vitreoretinal Division, King Khaled Eye Specialist Hospital, Riyadh 12211, Saudi Arabia; doaamilibari@gmail.com (D.M.); snowilaty@kkesh.med.sa (S.R.N.); 2 Department of Ophthalmology, King Abdullah Medical City, Makkah 24211, Saudi Arabia; 3 Ocular Genetics Services, King Khaled Eye Specialist Hospital, Riyadh 12211, Saudi Arabia

**Keywords:** Bardet–Biedl syndrome, BBSome, chaperonin complex, ciliopathy, cone–rod dystrophy, polydactyly, retinitis pigmentosa

## Abstract

The retinal features of Bardet–Biedl syndrome (BBS) are insufficiently characterized in Arab populations. This retrospective study investigated the retinal features and genotypes of BBS in Saudi patients managed at a single tertiary eye care center. Data analysis of the identified 46 individuals from 31 families included visual acuity (VA), systemic manifestations, multimodal retinal imaging, electroretinography (ERG), family pedigrees, and genotypes. Patients were classified to have cone–rod, rod–cone, or generalized photoreceptor dystrophy based on the pattern of macular involvement on the retinal imaging. Results showed that nyctalopia and subnormal VA were the most common symptoms with 76% having VA ≤ 20/200 at the last visit (age: 5–35). Systemic features included obesity 91%, polydactyly 56.5%, and severe cognitive impairment 33%. The predominant retinal phenotype was cone–rod dystrophy 75%, 10% had rod–cone dystrophy and 15% had generalized photoreceptor dystrophy. ERGs were undetectable in 95% of patients. Among the 31 probands, 61% had biallelic variants in BBSome complex genes, 32% in chaperonin complex genes, and 6% had biallelic variants in *ARL6*; including six previously unreported variants. Interfamilial and intrafamilial variabilities were noted, without a clear genotype–phenotype correlation. Most BBS patients had advanced retinopathy and were legally blind by early adulthood, indicating a narrow therapeutic window for rescue strategies.

## 1. Introduction

Inherited retinal disorders (IRDs) comprise a heterogeneous group of diseases that manifest with or without systemic associations. Some IRDs are due to perturbations of the development and maintenance of the photoreceptor cilium and other primary cilia in the body. Bardet–Biedl syndrome (BBS) (MIM 209900) is an autosomal recessive ciliopathy and is the second most common form of syndromic retinopathy after Usher syndrome [1]. The diagnosis of BBS is established if the following criteria are met: (1) the presence of four primary features or (2) the presence of three primary features with at least two secondary features [2]. The primary features are retinal dystrophy, truncal obesity, postaxial polydactyly, hypogonadism, cognitive impairment, and renal anomalies. The secondary features are developmental delay, ataxia, poor coordination, short stature, speech disorder, behavioral abnormalities, strabismus, cataract, astigmatism, dental abnormalities, high arched palate, craniofacial dysmorphism, brachydactyly, syndactyly, lower limb spasticity, reproductive abnormalities, diabetes mellitus, cardiovascular anomalies, and hepatic fibrosis [2]. 

Pathogenic variants in more than twenty genes have been associated with BBS [3]. BBS genes encode for proteins that play a crucial role in the BBSome or its chaperonin complex, which are involved in the ciliary biogenesis, regulation, and vesicular transport [4]. Most BBS cases are associated with perturbations of the BBSome complex: *BBS1*, *BBS2*, *BBS4*, *BBS5*, *BBS7*, *TTC8*, *BBS9*, and *BBIP1*, and less frequently with the genes encoding the BBS chaperonin complex: *MKKS*, *BBS10* and *BBS12* [4,5,6,7]. The other BBS genes are rare [7,8]. 

Previous studies described the clinical and molecular features of BBS in various endogamous and exogamous populations, but such data are sparse in the Arab population [1,9,10]. The present study expands the spectrum of BBS mutations in the Saudi population, includes previously unreported variants, and describes the associated retinal phenotypes.

## 2. Methods

This is a retrospective study of 46 individuals, from 31 families, who harbored biallelic variants in known BBS genes and were reviewed at King Khaled Eye Specialist Hospital (KKESH), a tertiary eye care center in Riyadh, Saudi Arabia, from 2007 to 2019. Institutional Review Board (IRB)/Ethics Committee approval at KKESH was obtained (IRB: RP1933-R) and the study adhered to the tenets of the declaration of Helsinki. 

Informed consent for clinical genetic testing was obtained from all participants. Data analyzed included: the earliest visual symptoms, age at onset of visual symptoms and last visit, sex, visual acuity at presentation and at last clinic visit, reported systemic features, and family history. All patients underwent full ophthalmic examination including Snellen VA, slit lamp biomicroscopy, and dilated fundoscopy. The retinal structure was evaluated with multimodal imaging: fundus images (Topcon, Tokyo, Japan; and Optos TM, Dunferline, Scotland, UK), widefield medium wavelength (532 nm) fundus autofluorescence (FAF, on the Optos machine), spectral-domain optical coherence tomography (OCT, Heidelberg Engineering, Heidelberg, Germany). Full-field electroretinography (ERG, Nicolet Biomedical Instruments, Madison, WI, USA; and Roland Consult, Brandenburg an der Havel, Germany) was performed using a protocol that was modified from the International Society for Clinical Electrophysiology of Vision Standards, as previously described [11].

**Figure 1:**
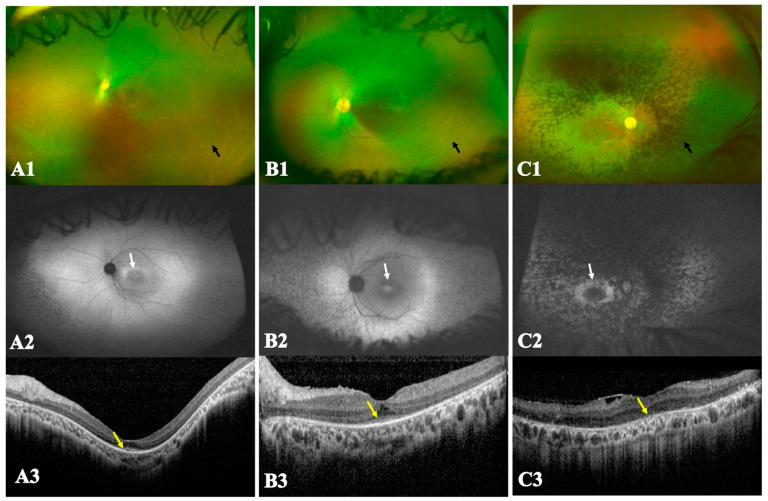
Ultra-widefield color fundus images, fundus autofluorescence (FAF) and optical coherence tomography (OCT) of the three retinal phenotypes: rod–cone dystrophy (RCD), cone–rod dystrophy (CRD) and generalized photoreceptor involvement. (**A**) (**A1**,**A2**,**A3**) An example of RCD; (**A1**) color photo showing widespread retinal alterations, vascular attenuation and multiple hypopigmented small patches in the mid-periphery (black arrow); (**A2**) FAF revealing an annulus of increased AF at the macula (white arrow); the hypopigmented patches in (**A1**) co-localize with hypo-autofluorescent patches in the mid-periphery indicating retinal atrophy; (**A3**) OCT depicting relatively spared sub-foveal ellipsoid zone (EZ) (yellow arrow). (**B**) (**B1**,**B2**,**B3**) An example of CRD; (**B1**) color image showing widespread retinal alterations with widespread patchy hypopigmentation (black arrow), note the relatively milder vascular attenuation compared to (**A**), and dull foveal reflex; (**B2**) FAF shows a patch of increased signal at the macula surrounded by reduced signal (white arrow); there is widespread patchy hypo-AF in the mid-periphery; (**B3**) OCT showing sub-foveal hyporeflective EZ which tapers abruptly at the edges of the fovea with loss of the outer nuclear layer (yellow arrow). (**C**) (**C1**,**C2**,**C3**) An example of generalized photoreceptor involvement; (**C1**) color image showing bone spicule-like pigmentation extending from the vascular arcades to the periphery (black arrow), vascular attenuation and macular atrophy; (**C2**) FAF revealed diffuse hypo-AF signal in the mid-peripheral retina and macula and a distinct macular annulus of retained signal surrounding a patch of signal loss (white arrow); (**C3**) OCT depicted an absent EZ (yellow arrow), epiretinal membrane and severe laminar disorganization.

Classification of FAF images, based on the patterns deduced from the macular AF changes, was performed jointly by two retina specialists (DM and RB) and was reviewed independently by a third retina specialist (SRN); in the case of discrepancy, RB reclassified the image(s). Since the ERGs were undetectable in 34 patients, and assuming that most patients with cone–rod dystrophy (CRD) would manifest with early severe maculopathy and foveal involvement, the retinal phenotype was classified as (1) rod–cone dystrophy (retinitis pigmentosa): if the VA at presentation was better than 20/200, with an annulus of increased AF at the macula and a sub-foveal EZ depicted on OCT (Figure 1(A1,A2,A3)); (2) CRD: if the VA at presentation was less than or equal to 20/200—used as a measure for foveal dysfunction, and the macular AF showed a pattern reminiscent of a bull’s eye lesion with disrupted or hyporeflective ellipsoid zone (EZ) on OCT (Figure 1(B1,B2,B3)); (3) generalized photoreceptor dystrophy if the VA was less than 20/200, the AF did not show a distinct macular annulus of increased signal, and the OCT showed an absent or markedly disrupted EZ (Figure 1(C1,C2,C3)) [12,13]. 

Genetic testing was performed at two clinical laboratories using next-generation sequencing for the following retinal panels (Figure S1): autosomal recessive retinitis pigmentosa, BBS, cone–rod dystrophy (CRD), cone dystrophy (CD), and macular dystrophy as previously described [14]. Classification of variants, based on the laboratory reports, was also scrutinized using Varsome 11.8 [15]; previously unreported variants were classified according to the ACMG guidelines [16]. 

## 3. Results

### 3.1. Patient Characteristics and Clinical Features

Seventy-one individuals with a clinical diagnosis of BBS were identified; twenty-five of them were not molecularly characterized and were excluded. Forty-six patients from thirty-one families were included. A summary of their demographic data is given in Table 1. The age range was 5–35 years (median: 19 years). Twenty-seven patients were males (59%).

Poor night vision and subnormal visual acuity were the initial symptoms and noted during the first decade of life in 70% of the cases. Nystagmus was noted at presentation in seventeen patients (37%). Initial VA was 20/200 (1.00 logMAR) or less in the better seeing eye in 27/46 patients (58.7%), better than 20/200 in 12/46 (26.1%), and not measured in 7 patients who were able to fixate and follow but had limited cooperation. Of the 12 with initial VA better than 20/200, vision deteriorated to 20/200 or worse in five patients (41.6%; duration 1–10 years, mean 6.4). At the last clinic visit, the VA was 20/200 or worse in the better-seeing eye in 35/46 patients (76%) and better than 20/200 in 9/46 (19.6%); the remaining two patients were able to fixate and follow. The most frequent systemic features were obesity 42/46 (91.3%), polydactyly 26/46 (56.5%), and severe cognitive disability 15/46 (32.6%).

### 3.2. Multimodal Imaging and Electroretinographic Features

**Figure 2:**
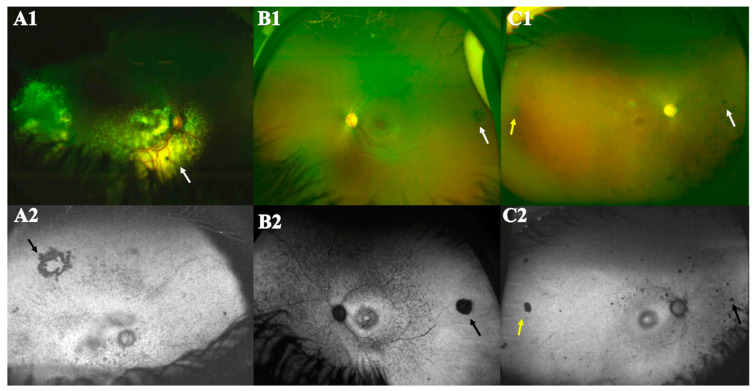
Ultra-widefield color fundus images and fundus autofluorescence (FAF) of three different patients with distinct features. (**A**) Patient 28B has Coats’-like picture; (**A1**) color image showing macular and peripheral exudation and telangiectatic blood vessels (white arrow); (**A2**) FAF revealed patches of decreased AF inferior to the macula and in the midperiphery with loss of AF at the macular center, a ring of loss of AF (cryotherapy mark) is noted supero-temporally (black arrow). (**B**) Patient 30A has cone–rod dystrophy and congenital hypertrophy of the retinal pigment epithelium (CHRPE) lesion temporally; (**B1**) color image showing vascular attenuation, and a bull’s eye lesion at the macula; a round pigmented lesion comprising lacunae (CHRPE, white arrow); (**B2**) FAF revealed round area of decreased AF temporally (black arrow), numerous small patches of decreased AF at the midperiphery and anterior to the arcades with a central patch of increased AF at the macular center, surrounded by an annulus of decreased AF. (**C**) Patient 18B has nummular pigmentation; (**C1**) color image showing retinal atrophy, severe vascular attenuation, bone spicules and nummular pigmentation in the nasal midperiphery (white arrow), a CHRPE lesion temporally (yellow arrow) and a bull’s eye lesion at the macula. (**C2**) FAF revealed patches and nummular dots of decreased AF at the midperiphery (black arrow), round area of decreased AF temporally (yellow arrow) and adjacent to the arcades with a central patch of increased AF at the macular center, surrounded by an annulus of decreased AF.

The most frequent fundus features were retinal pigmentary alterations (84%), vascular attenuation (95.5%), mid-peripheral bone spicule-like pigmentation (55.5%) and macular atrophy (24%) (Figure S2, Table 2). Other clinical findings included Coats’-like picture (2.2%) [patient 28B] (Figure 2(A1,A2)), congenital hypertrophy of the retinal pigment epithelium (2.2%) [patient 30A] (Figure 2(B1,B2)), and nummular pigmentation (11%) [patients: 11, 18A, 18B, 28C, 28D] (Figure 2(C1,C2)). Fundus autofluorescence was available for 43 patients (Figure S3), one patient (20A) had cataract and was therefore excluded from the macular AF analysis. The macular AF pattern was classified as the following: (1) bull’s eye lesion: hyper-autofluorescence at the macular center, with or without an annulus of decreased AF (Figure S3: 2A; 3B; 5C; 8A; 8B; 9; 11; 12; 13B; 13C; 14; 15; 16; 17; 18A; 18B; 19; 20B; 21; 22; 24; 25; 26; 28A; 28C; 29; 30A); (2) atrophic maculopathy: geographic or nummular loss of AF at the macular center (Figure S3: 1; 2B; 3A; 6; 7; 10; 23; 27A; 27B; 28B; 28D); (3) perifoveal annulus of increased AF, with unremarkable central macular signal (Figure S3: 5A and 13A); (4) the remaining two subjects had a patch of decreased AF at the macular center (Figure S3: 30B, 31).

Optical coherence tomography scans were available for 41 patients (Figure S4a). To characterize the phenotype, the pattern of retinal lamination was graded according to severity (Figure S4b): (1) unremarkable lamination (3/41 patients, 7.3%); (2) partially disorganized: disrupted retinal lamination with variability within the same scan (23/41; 56.1%); (3) severely disorganized: indistinct lamination (15/41, 36.6%). Additionally, the ellipsoid zone (EZ) was described as either absent (25/41 patients, 60.9%), or disrupted (16/41 patients, 39%).

Thirty-six patients underwent full field ERG examination. Scotopic and photopic responses were undetectable in 34 patients (94.5%; Table 2). One patient showed a rod–cone dystrophy pattern (Figure S5—patient 5B), and another showed a cone–rod dystrophy pattern (Figure S5—patient 13B).

Because most patients had undetectable ERGs, the patients in this cohort were classified according to the VA, the macular AF and OCT findings as described in the Methods section. Of the 40 patients who had AF and OCT images, thirty (75%) were classified to have cone–rod dystrophy, six (15%) had generalized photoreceptor dystrophy, and four (10%) had rod–cone dystrophy (Table 2).

### 3.3. Molecular Genetics

The probands from 31 families were genotyped (Figure S6). Twenty patients were simplex cases and underwent genotyping and phenotyping. For families with multiple affected members, genotyping was carried out only for the probands, but the clinical data were analyzed for all the affected individuals who presented to the IRD clinic. Nine probands (29%) had variants in *BBS4*, six (19.4%) in *MKKS*, four (13%) in *BBS1*, and three (9.7%) in *BBS5*. Eight probands had variants in one of the following genes: *BBS9*, *BBS10*, *BBS12*, *ARL6* (6.5% each), and one proband had variants in *BBS2* (3.2%) (Table 3). Nineteen probands (61%) harbored a mutation in one of BBSome complex genes, ten (32%) harbored a mutation in one of chaperonin complex genes (Table 3). Two probands (6%) were homozygous for variants in *ARL6*.

Family pedigrees were available for 23 of the 31 probands (Figure S6). Twenty-two probands (71%) came from consanguineous families, and harbored homozygous variants in one of the BBS genes, except for family 23 where the proband was heterozygous for two variants in *BBS4* (Table 3). Six probands came from non-consanguineous families, four harbored homozygous variants in *BBS1, BBS10*, and *MKKS*; and two harbored two heterozygous variants in *BBS2* and *BBS1.* Three probands came from non-consanguineous families, but the parents originated from the same tribe, and they harbored homozygous variants in *BBS1*, *BBS4*, and *BBS12*.

Five probands had previously unreported variants. Proband 8 harbored a homozygous splice-site variant in *BBS9* (NM_198428): c.617+3A>C. This variant was not reported in gnomAD, ClinVar or LOVD (PM2) [17,18,19], and was predicted to break the doner splice-site (Human Splicing Finder Pro [20,21]: MaxEnt Donor site at position chr7:33257408, variation: −107.81%) (PP3) [16], and the phenotype was in keeping with the diagnosis of BBS (PP4). This variant was classified as a variant of uncertain significance. Proband 9 harbored a homozygous splice-site variant in *BBS5* (NM_152384): c.900+1G>A, which was classified as pathogenic. Proband 17 had homozygous in-frame deletion of three nucleotides in *BBS10* (NM_024685): c.1195_1197del, p.(Leu399del). This variant was classified as likely pathogenic based on the following criteria: (1) it changes the protein length (PM4); (2) it is predicted by in silico tools to have a significant impact on the protein (PP3); (3) the phenotype is in keeping with the diagnosis of BBS (PP4); and (4) PM2. In addition, three pathogenic variants were detected in *BBS4* (NM_033028): c.1159G>T, p.(Glu387*), c.262delG, p.(Glu88Asnfs*54), c.1311_1312insT, p.(Lys438*)–probands 20 and 23 (Table 3).

Recurrent variants were identified: *BBS1*: c.951+58C>T p.(Gly318Valfs*61), in four probands; six probands had a recurrent variant in *BBS4*: c.157-2A>G, two probands had a variant in *BBS5*: c.966dupT p.(Ala323Cysfs*57); five probands had a recurrent variant in *MKKS*: c.116C>T p.(Pro39Leu); and two probands had the same variant in *BBS12*: c.787dupT p.(Tyr263Leufs*4) (Table 3).

**Table 3:** Molecular data of the 31 probands (novel mutations identified in this study are marked with an asterisk [*]).

Proband	Gene Group	Gene	Allele 1	Allele 2	Pathogenicity
1	Others	*ARL6* (NM_001278293)	c.431C>T p.(Ser144Phe) [9,22,23]	c.431C>T p.(Ser144Phe)	Likely pathogenic
2	BBSome	*BBS1* (NM_024649)	c.124+1G>A [24,25]	c.951+58C>T p. (Gly318Valfs*61) [26]	Pathogenic
3	Chaperonin complex	*BBS10* (NM_024685)	c.924G>T p.(Leu308Phe) [27]	c.924G>T (p.Leu308Phe)	Pathogenic
4	BBSome complex	*BBS4* (NM_033028)	c.157-2A>G [9,28]	c.157-2A>G	Pathogenic
5	BBSome complex	*BBS4* (NM_033028)	c.157-2A>G [9,28]	c.157-2A>G	Pathogenic
6	BBSome complex	*BBS4* (NM_033028)	c.157-2A>G [9,28]	c.157-2A>G	Pathogenic
7	BBSome complex	*BBS5* (NM_152384)	c.966dupT: p.(Ala323Cysfs*57) [29]	c.966dupT: p.(Ala323Cysfs*57)	Pathogenic
8	BBSome complex	*BBS9* (NM_198428)	c.617+3A>C [*]	c.617+3A>C	VUS
9	BBSome complex	*BBS5* (NM_152384)	c.900+1G>A [*]	c.900+1G>A	Pathogenic
10	Chaperonin complex	*MKKS* (NM_170784)	c.116C>T p.(Pro39Leu) [24,25]	c.116C>T p.(Pro39Leu)	Likely pathogenic
11	Others	*ARL6* (NM_001278293)	c.362G>A p.(Arg121His) [30,31]	c.362G>A p.(Arg121His)	Likely pathogenic
12	Chaperonin complex	*MKKS* (NM_170784)	c.295T>C p.(Cys99Arg) [32,33]	c.295T>C p.(Cys99Arg)	Likely pathogenic
13	Chaperonin complex	*MKKS* (NM_170784)	c.116C>T p.(Pro39Leu) [24,25]	c.116C>T p.(Pro39Leu)	Likely pathogenic
14	BBSome complex	*BBS4* (NM_033028)	c.157-2A>G [9,28]	c.157-2A>G	Pathogenic
15	BBSome complex	*BBS4* (NM_033028)	c.1106+2T>A [34,35]	c.1106+2T>A	Pathogenic
16	BBSome complex	*BBS4* (NM_033028)	c.157-2A>G [9,28]	c.157-2A>G	Pathogenic
17	Chaperonin complex	*BBS10* (NM_024685)	c.1195_1197delCTT (p. Leu399del) [*]	c.1195_1197delCTT (p. Leu399del)	Likely pathogenic
18	BBSome complex	*BBS2* (NM_031885)	c.471G>A p.(Thr157=) [36]	c.944G>A p.(Arg315Gln) [37]	Likely pathogenic
19	BBSome complex	*BBS1* (NM_024649)	c.951+58C>T p.(Gly318Valfs*61) [26]	c.951+58C>T p.(Gly318Valfs*61)	Pathogenic
20	BBSome complex	*BBS4* (NM_033028)	c.1159G>T p.(Glu387*) [*]	c.1159G>T p.(Glu387*)	Pathogenic
21	BBSome complex	*BBS1* (NM_024649)	c.951+58C>T p.(Gly318Valfs*61) [26]	c.951+58C>T p.(Gly318Valfs*61)	Pathogenic
22	BBSome complex	*BBS4* (NM_033028)	c.157-2A>G [9,28]	c.157-2A>G	Pathogenic
23	BBSome complex	*BBS4* (NM_033028)	c.262delG p.(Glu88Asnfs*54) [*]	c.1311_1312insT p.(Lys438*) [*]	Pathogenic
24	BBSome complex	*BBS9* (NM_198428)	c.832C>T p.(Arg278*) [38]	c.832C>T p.(Arg278*)	Pathogenic
25	Chaperonin complex	MKKS (NM_170784)	c.116C>T p.(Pro39Leu) [24,25]	c.116C>T p.(Pro39Leu)	Pathogenic
26	Chaperonin complex	BBS12 (NM_001178007)	c.787dupT p.(Tyr263Leufs*4) [25]	c.787dupT p.(Tyr263Leufs*4)	Pathogenic
27	BBSome complex	BBS1 (NM_024649)	c.951+58C>T p.(Gly318Valfs*61) [26]	c.951+58C>T p.(Gly318Valfs*61)	Pathogenic
28	Chaperonin complex	BBS12 (NM_001178007)	c.787dupT p.(Tyr263Leufs*4) [25]	c.787dupT p.(Tyr263Leufs*4)	Pathogenic
29	Chaperonin complex	MKKS (NM_170784)	c.116C>T p.(Pro39Leu) [24,25]	c.116C>T p.(Pro39Leu)	Pathogenic
30	Chaperonin complex	MKKS (NM_170784)	c.116C>T p.(Pro39Leu) [24,25]	c.116C>T p.(Pro39Leu)	Pathogenic
31	BBSome complex	BBS5 (NM_152384)	c.966dupT: p.(Ala323Cysfs*57) [29]	c.966dupT: p.(Ala323Cysfs*57)	Pathogenic

### 3.4. Genotype–Phenotype Correlation

Clinical classification of retinopathy showed a continuum where the predominantly affected photoreceptor cell type was not always determined. All but four patients had syndromic features (Table 1). The four probands with apparently non-syndromic CRD or generalized photoreceptor degeneration had biallelic variants in *BBS4* (proband 6), *ARL6* (proband 11) and *BBS6* (probands 12 and 25) (Table 1 and Table 3).

The retinal phenotype in three patients (5A, 5B, 13A) was classified as rod–cone dystrophy. Family 5 was homozygous for a variant in *BBS4*: c.157-2A>G; this variant was identified in five other families (4, 6, 14, 16, 22) with cone–rod dystrophy or generalized photoreceptor degeneration. 

The proband from family 13 was homozygous for a variant in *MKKS*: c.116C>T, p.(Pro39Leu); while one sibling (13A) manifested with rod–cone dystrophy, the other two (13B and 13C) had CRD. Family 30 harbored the same variant in *MKKS*, with one member (30B) manifesting with rod–cone dystrophy, while the other sibling (30A) manifested with CRD. 

There was no correlation between the genotype and the severity of central visual loss in this study. Nine patients had VA > 20/200 in the better seeing eye on the last clinical examination. The age range was 9–20 years (median: 16 years) (Table 2 and Table 3). These patients harbored biallelic variants in genes encoding components of the BBSome, as well as the chaperonin complex: *BBS1*, *BBS4*, *BBS5*, *BBS9*, *BBS12*, *MKKS*. Twenty-seven patients had visual acuity ≤ 20/200 at presentation (age 6–32 years, median: 20 years). Those patients harbored biallelic variants in the same genes found in the former group in addition to *ARL6*, *BBS10*, *BBS2*. 

## 4. Discussion

In this study, the retinal and main systemic manifestations of BBS were described in a cohort of Saudi patients, managed at the country’s largest tertiary eye care center. We also identified six previously unreported variants in four BBS genes. 

Most patients in this study had advanced retinal degeneration such that phenotypic classification to have either rod–cone dystrophy or CRD was not feasible based on ERG, as previously reported [26,39]. Other parameters that aid in phenotyping are clinical history and retinal imaging. Although the first two symptoms commonly reported were nyctalopia and reduced visual acuity, due to foveal involvement, the latter suggests that the foveal cones are particularly vulnerable to dysfunctional BBS proteins. Moreover, information can be gained from retinal imaging. For example, the distribution of macular AF changes, such as the parafoveal ring of increased signal with normal signal in the middle, as seen in rod–cone dystrophy, or altered central signal as seen in cases of cone–rod dystrophy is better defined than mid-peripheral AF where the signal can be either indistinct (featureless) or lost, due to deep retinal changes or intra-retinal pigment migration and loss of the retinal pigment epithelium, respectively [12,13]. Both mid-peripheral changes can occur in rod–cone dystrophy or CRD. Macular OCT is also useful in assessing the integrity of the foveal ellipsoid zone and assessing the degree of loss of lamination, due to retinal remodeling [40]. The classifications proposed in this study suggest that only a minority of patients had typical retinitis pigmentosa. This differs from other reports of large cohorts, which showed, based on ERG recordings, a rod–cone dystrophy pattern [3]. This difference is due to our reliance on retinal imaging, highlighting a discrepancy between retinal features and the retinal mass responses on ERG. 

Four patients in this study, carrying four different homozygous variants in *BBS4*, *ARL6*, and *MKKS* genes, were found to have no obvious syndromic features. Previously, variants in *C8orf37* [8], *ARL6* [41,42], and *BBS8* [43] were associated with non-syndromic retinopathy [44]. The absence of syndromic features could be due to the presence of retina-specific isoforms of the same gene, or the involvement of amino acids that are crucial for the photoreceptor function but have no clinically significant impact on the cilia in other organs.

The most common genes in this study were *BBS4* (29%) and *MKKS* (19.4%). This finding differs from other national studies from a general hospital setting where *ARL6*, *BBS1*, and *BBS2* were the most frequently mutated genes [10,22]. Twenty-five patients in this study were excluded as their genotypes were not available, and this might have contributed to this difference. Additionally, patients with significant visual loss, particularly those with non-syndromic retinopathy or only subtle features, are usually diagnosed with BBS by ophthalmologists, whilst those with prominent systemic features present first to general hospitals. Internationally, the most frequently mutated BBS genes were *BBS1* and *BBS10* [4,38,45].

The high prevalence of homozygous variants in this study offers an opportunity to assess the effect of these variants on the retina and aids in the diagnosis of patients who may harbor one of these variants *in trans* with a novel variant. Intrafamilial variability of systemic manifestations of BBS was documented in the literature; however, little is known about variability of the retinal phenotype within the same family [46,47]. As previously reported, there was no clear genotype–phenotype correlation in our cohort [45,48,49,50]. Additionally, we identified a recurrent variant in *BBS4*: c.157-2A>G causing both CRD and rod–cone dystrophy. Similarly, a recurrent variant in *MKKS*: c.116C>T, p.(Pro39Leu) caused CRD and rod–cone dystrophy in different individuals. As observed by others, variants in the same BBS gene have been reported to give rise to either CRD or retinitis pigmentosa [26,51].

Inherited retinal disorders were recently ranked at the top of the causes of blind registration in the working age group in developed countries [52]. Given that the median age in this study was 19 years, and 76% of the patients were legally blind at the last clinic visit, it is reasonable to conclude that BBS would have a stronger socio-economic impact compared to non-syndromic IRDs, as patients are also affected by other comorbidities. Future therapeutic trials for patients with BBS should consider targeting the systemic manifestations as well as multiple cell types in the retina since it has been suggested that BBS proteins are also expressed in other retinal cell types [53,54]. The early presentation and rapid progression of visual loss in BBS patients impose a narrow window of opportunity for novel therapeutic interventions such as gene rescue; therefore, other approaches such as optogenetics and visual rehabilitation would be more suitable for advanced retinopathy. Premarital screening in highly consanguineous populations, genetic counselling, and a multidisciplinary approach remain the standard of care for BBS patients.

In conclusion, this study has the largest Middle Eastern cohort to depict BBS as a severe form of retinopathy in our population. Additionally, the study added six previously unreported variants to the genetic spectrum of BBS. Due to the retrospective nature of the current study, the depth of systemic phenotyping was limited, which may have led to underreporting of BBS systemic manifestations.

## Figures and Tables

**Table 1:** Demographic data and clinical features of the patients. Abbreviations: M: male, F: female.

Patient	Age	Sex	Age of Onset of Visual Symptoms	Earliest Visual Symptoms	Systemic Features	Consanguinity
1	23	M	Early childhood	Nystagmus and poor night vision	Obesity and polydactyly	1st cousins
2A	12	F	Early childhood	Poor night vision	Obesity	No
2B	22	F	Early childhood	Nystagmus and poor night vision	Obesity	No
3A	26	M	Early childhood	Nystagmus and poor night vision	Obesity, polydactyly and cardiac disease	No
3B	28	F	Early infancy	Nystagmus and poor night vision	Obesity and polydactyly	No
4A	19	M	Early childhood	Poor day and night vision	Obesity and polydactyly	same tribe
4B	24	F	Early childhood	Poor day and night vision	Obesity and polydactyly	same tribe
5A	13	F	Early infancy	Nystagmus, poor night vision and poor navigation	Obesity and cognitive disability	1st cousins
5B	4	F	Early infancy	Nystagmus, poor night vision and poor navigation	Obesity and polydactyly	1st cousins
5C	11	M	Early infancy	Nystagmus and poor night vision	Obesity, polydactyly and hypothyroidism	1st cousins
6	33	M	Early childhood	Poor night vision	None	1st cousins
7	15	M	Early infancy	Nystagmus	Obesity, polydactyly renal impairment and cognitive disability	1st cousins
8 A	13	M	Early infancy	Poor night vision	Obesity, polydactyly and hypothyroidism	1st cousins
8B	9	F	Early childhood	Poor night vision	Obesity and brachydactyly	1st cousins
9	15	M	Early childhood	Poor night vision	Obesity, polydactyly, renal impairment and hypogonadism	1st cousins
10	33	M	Early childhood	Poor night vision and day vision	Obesity and polydactyly	1st cousins
11	28	F	Adulthood	Poor day vision	None	1st cousins
12	22	M	Early childhood	Poor night vision	None	1st cousins
13A	15	M	Early infancy	Poor navigation	Obesity and polydactyly	No
13B	16	F	Early infancy	Poor navigation	Obesity and polydactyly	No
13C	11	M	Early infancy	Nystagmus	Obesity and polydactyly	No
14	10	M	Early infancy	Nystagmus and poor night vision	Obesity and cognitive disability	1st cousins
15	33	M	Early childhood	Poor day vision	Obesity and cognitive disability	1st cousins
16	21	F	Early childhood	Poor day and night vision	Obesity, polydactyly and cognitive disability	1st cousins
17	28	M	Early childhood	Poor day and night vision	Obesity and polydactyly	No
18A	35	M	Early childhood	Poor day and night vision	Obesity and polydactyly	No
18B	30	M	Early childhood	Poor day and night vision	Obesity	No
19	20	M	Early childhood	Poor navigation Poor day and night vision	Obesity and polydactyly	Same tribe
20A	31	F	Early childhood	Poor night vision	Obesity, polydactyly, renal impairment, cognitive disability and splenomegaly	1st cousins
20B	22	F	Early infancy	Nystagmus, poor day and night vision	Obesity and renal impairment	1st cousins
21	34	F	Early childhood	Poor day vision	Obesity and cognitive disability	No
22	6	M	Early infancy	Nystagmus	Obesity	1st cousins
23	34	F	Adulthood	Poor night vision	Obesity	2nd cousins
24	17	M	Early childhood	Nystagmus, poor day and night vision	Obesity, polydactyly, cognitive disability and hypogonadism	1st cousins
25	15	F	Early childhood	Nystagmus, poor day and night vision	None	1st cousins
26	19	F	Early childhood	Poor night vision	Obesity	1st cousins
27A	30	F	Early childhood	Poor day and night vision	Obesity, polydactyly and benign lung tumor	1st cousins
27B	24	M	Early childhood	Poor day and night vision	Obesity, short stature	1st cousins
28A	28	F	Early childhood	Nystagmus, poor day and night vision	Obesity, polydactyly and cognitive disability	Same tribe
28B	19	M	Early childhood	Nystagmus, poor day and night vision	Obesity, cognitive disability and hypothyroidism	Same tribe
28C	34	M	Early childhood	Poor day and night vision	Obesity, polydactyly and syndactyly cognitive disability	Same tribe
28D	30	F	Early childhood	Poor day and night vision	Obesity, diabetes, cognitive disability, renal impairment and hypothyroidism	Same tribe
29	17	M	Early childhood	Poor day and night vision	Obesity and polydactyly	2nd cousins
30A	13	M	Early childhood	Nystagmus, poor night vision	Obesity, polydactyly, syndactyly, cognitive disability and delay speech	1st cousins
30B	8	M	Early childhood	Esotropia and poor night vision	Obesity, polydactyly, and cognitive disability	1st cousins
31	10	M	Early childhood	Exotropia, nystagmus and poor day and night vision	Obesity, cognitive disability and hypogonadism	1st cousins

**Table 2:** Ocular features, multimodal images and electrophysiological findings of the patients; Abbreviations: (VA: visual acuity, OCT: optical coherence tomography, FAF: fundus autofluorescence, ERG: electroretinography, UD: undetectable, NA: not available).

Patient	Age	VA at Presentation (Age)	VA at Last Visit (Age)	Fundus Images	FAF Finding	Phenotype	OCT Finding	ERG
1	23	20/300 (14Y)	HM OU (21Y)	Retinal atrophy, vascular attenuation, midperipheral bone spicules and macular atrophy	Patches of decreased AF around the arcades with patchy loss of AF at the macula	CRD	Partial disorganization of retinal lamination with loss of EZ.	NA
2A	12	20/100 and 20/400 (5 Y)	20/300 and 20/400 (13 Y)	Retinal atrophy, vascular attenuation and macular pigment alteration.	Patches of decreased AF at the midperiphery with a central patch of increased AF at the macula surrounded by an annulus of decreased AF.	CRD	Partial disorganization of retinal lamination with loss of EZ.	UD
2B	23	LP OU (23Y)	LP OU (23Y)	Retinal atrophy, vascular attenuation, midperipheral bone spicules and macular atrophy.	Patches of decreased AF of the retina with patchy loss of AF at the macula.	CRD	Partial disorganization of retinal lamination with disrupted EZ.	UD
3A	26	1/200 OU (19 Y)	1/200 OU (25Y)	Vascular attenuation midperipheral bone spicules and macular atrophy.	Patches of decreased AF at the midperipheral retina with patchy loss of AF at the macula.	Generalized photoreceptor involvement	Severe disorganization of retinal lamination with loss of EZ.	UD
3B	28	CF OU (14 Y)	HM OU (25)	Vascular attenuation, midperipheral bone spicules and macular pigment alteration.	Patches of decreased AF at the midperiphery with a central patch of increased AF at the macular surrounded by an annulus of decreased AF.	Generalized photoreceptor involvement	Severe disorganization of retinal lamination with loss of EZ.	UD
4A	19	CF OU (12 Y)	CF OU (12Y)	Retinal atrophy and vascular attenuation.	NA	Not classified	Partial disorganization of retinal lamination with disrupted EZ.	UD
4B	24	HM OD and 2/200 (18 Y)	HM OD and 2/200 (18 Y)	Retinal atrophy, vascular attenuation and macular atrophy.	NA	Not classified	NA	UD
5A	13	20/100 OU (9 Y)	20/100 and 20/60 (13 Y)	Retinal atrophy and vascular attenuation.	Patches of decreased AF with perifoveal annulus of increased AF.	RCD	Partial disorganization of retinal lamination with disrupted EZ.	UD
5B	4	F and F (1Y)	F and F (4 Y)	NA	NA	Not classified	NA	Reduced photonic and scotopic responses (Figure S5)
5C	11	20/100 and 20/200 (9Y)	20/100 and 20/200 (11 Y)	Retinal atrophy and vascular attenuation.	Patches of decreased AF at the midperiphery with perifoveal annulus of increased AF.	RCD	Partial disorganization of retinal lamination with disrupted EZ.	UD
6	33	LP OU (27 Y)	LP OU (34 Y)	Vascular attenuation, Midperipheral bone spicules, and macular atrophy.	Patches of decreased AF in the midperiphery with patchy loss of AF at the macula.	Generalized photoreceptor involvement.	Partial disorganization of retinal lamination, with loss of EZ.	UD
7	15	HM/LP (12Y)	HM/LP (16 Y)	Retinal atrophy, vascular attenuation, midperipheral bone spicules and macular atrophy.	Patches of decreased AF in the midperiphery with patchy loss of AF at the macula.	Generalized photoreceptor involvement.	Severe disorganization of retinal lamination with loss of EZ.	UD
8A	13	F and F (8 Y)	20/100 and 20/80 (14Y)	Retinal atrophy, vascular attenuation and macular pigment alteration.	Patches of decreased AF in the midperiphery with a central patch of increased AF at the macula surrounded by an annulus of decreased AF.	CRD	Unremarkable lamination with disrupted EZ.	NA
8B	9	F and F (4 Y)	20/80 and 20/100 (9 Y)	Retinal atrophy, vascular attenuation and bull’s eye maculopathy.	Patches of decreased AF in the midperiphery with a central patch of increased AF at the macula surrounded by an annulus of decreased AF.	CRD	Unremarkable lamination with disrupted EZ.	UD
9	15	20/100 OU (10Y)	20/100 OU (16Y)	Retinal atrophy, vascular attenuation, and bull’s eye maculopathy.	Patches of decreased AF at the midperiphery and around the arcades with a central patch of increased AF at the macula surrounded by an annulus of decreased AF.	CRD	Partial disorganization of retinal lamination with disrupted EZ.	UD
10	33	HM OU (30 Y)	HM OU (34Y)	Retinal atrophy, vascular attenuation, midperipheral bone spicules and macular atrophy.	Patches of decreased AF at the midperiphery and around the arcades with patchy loss of AF at the macula.	Generalized photoreceptor involvement.	Severe disorganization of retinal lamination with loss of EZ.	NA
11	28	2/200 (23Y)	2/200 (29Y)	Retinal atrophy, vascular attenuation, Scanty midperipheral bone spicules, scanty nummular pigmentations and bull’s eye maculopathy.	Patches and nummular dots of decreased AF at the midperiphery and around the arcades with a central patch of increased AF at the macula surrounded by an annulus of decreased AF.	CRD	Partial disorganization of retinal lamination with loss of EZ.	UD
12	21 FU 6 Y	20/100 and 20/125 (16 Y)	20/400 OU (22 Y)	Retinal atrophy, vascular attenuation, midperipheral bone spicules and bull’s eye maculopathy.	Patches of decreased AF at the midperiphery and around the arcades with a central patch of increased AF at the macula surrounded by an annulus of decreased AF.	CRD	Partial disorganization of retinal lamination with disrupted EZ.	UD
13A	15 FU 3 Y	20/70 OU (12 Y)	20/200 and 20/100 (15 Y)	Retinal atrophy	Perifoveal annulus of increased AF	RCD	Partial disorganization of retinal lamination with disrupted EZ.	UD
13B	16	2/200 (13 Y)	3/200 and 20/400 (16 Y)	Retinal atrophy, vascular attenuation, and bull’s eye maculopathy.	A central patch of increased AF at the macula surrounded by an annulus of decreased AF.	CRD	Partial disorganization of retinal lamination with disrupted EZ.	Reduced scotopic response and unrecordable photopic response. (Figure S5)
13C	10	CF OU (6 Y)	CF OU (10 Y)	Retinal atrophy, vascular attenuation, and macular pigment alteration.	A central patch of increased AF at the macula surrounded by an annulus of decreased AF.	CRD	Partial disorganization of retinal lamination with disrupted EZ.	UD
14	10	F and F (4 Y)	20/300 and 5/200 (11 Y)	Retinal atrophy, vascular and macular pigment alteration.	Patches of decreased AF in the midperiphery with a central patch of increased AF at the macula surrounded by an annulus of decreased AF.	CRD	Partial disorganization of retinal lamination with loss of EZ.	UD
15	33	HM and LP (28 Y)	HM and LP (34 Y)	Retinal atrophy, vascular attenuation, midperipheral bone spicules and bull’s eye maculopathy.	Patches of decreased AF in the midperiphery with a central patch of increased AF at the macula surrounded by an annulus of decreased AF.	CRD	Severe disorganization of retinal lamination, with loss of EZ.	NA
16	21	LP OU (17 Y)	LP OU (22Y)	Retinal atrophy, vascular attenuation, Scanty midperipheral bone spicules and bull’s eye maculopathy.	Patches of decreased AF in the midperiphery with a central patch of increased AF at the macula surrounded by an annulus of decreased AF.	CRD	Partial disorganization of retinal lamination with loss of EZ.	UD
17	28	1/200 and 20/300 (23Y)	1/200 and 20/300 (29 Y)	Retinal atrophy, vascular attenuation, Scanty midperipheral bone spicules and bull’s eye maculopathy.	Patches of decreased AF in the midperiphery with a central patch of increased AF at the macula surrounded by an annulus of decreased AF.	CRD	Partial disorganization of retinal lamination with loss of EZ.	UD
18A	35	HM OU (29 Y)	LP OU (35 Y)	Retinal atrophy, vascular attenuation, Scanty midperipheral bone spicule and macular pigment alteration.	Patches of decreased AF at the midperiphery and around the arcades with a central patch of increased AF at the macula surrounded by an annulus of decreased AF.	CRD	Severe disorganization of retinal lamination with loss of EZ.	UD
18B	30	20/400 and 20/300 (23 Y)	HM and 20/300 (30 Y)	Retinal atrophy, vascular attenuation, midperipheral bone spicules, scanty nummular pigmentations and macular pigment alteration.	Patches and nummular dots of decreased AF at the midperiphery and around the arcades with a central patch of increased AF at the macula surrounded by an annulus of decreased AF.	CRD	Partial disorganization of retinal lamination with loss of EZ.	UD
19	20	20/300 and 20/100 (18 Y)	20/100 and 20/100 (20 Y)	Retinal atrophy, vascular attenuation, midperipheral bone spicules and macular pigment alteration.	Midperipheral hypo autofluorescence patches, a central patch of increased AF at the macula surrounded by an annulus of decreased AF.	CRD	Severe disorganization of retinal lamination with loss of EZ.	UD
20A	31	HM OU (27 Y)	HM OU (30 Y)	Retinal atrophy, vascular attenuation, and scanty midperipheral bone spicules.	Patches of decreased AF in the midperiphery with we could not assess the macular AF features due cataract.	CRD	Severe disorganization of retinal lamination with loss of EZ.	UD
20B	22	LP OU (19 Y)	LP OU (22 Y)	Retinal atrophy, vascular attenuation and macular pigment alteration.	Patches of decreased AF in the midperiphery with a central patch of increased AF at the macula surrounded by an annulus of decreased AF.	CRD	Severe disorganization of retinal lamination with loss of EZ.	UD
21	34	LP OU (32 Y)	LP OU (34 Y)	Retinal atrophy, vascular attenuation, midperipheral bone spicules and bull’s eye maculopathy.	Patches of decreased AF in the midperiphery with a central patch of increased AF at the macula surrounded by an annulus of decreased AF.	CRD	Severe disorganization of retinal lamination with disrupted EZ.	NA
22	6	F andF (4 Y)	F andF (6Y)	Retinal atrophy and vascular attenuation and macular pigment alteration.	Patches of decreased AF in the midperiphery with a central patch of increased AF at the macula surrounded by an annulus of decreased AF.	CRD	Partial disorganization of retinal lamination with disrupted EZ.	UD
23	34	20/60 and 4/200 (23 Y)	20/300 OU (33 y)	Retinal atrophy, vascular attenuation, midperipheral bone spicules and macular atrophy.	Patches of decreased AF in the midperiphery with a patchy loss of AF at the macula.	CRD	Partial disorganization of retinal lamination with loss of EZ.	UD
24	17	CF OU (14 Y)	CF OU (18 Y)	Retinal atrophy and bull’s eye maculopathy.	A central patch of increased AF at the macula surrounded by an annulus of decreased AF.	CRD	Partial disorganization of retinal lamination with disrupted EZ.	UD
25	15	6/200 and 20/100 (7 Y)	20/300 AND 20/400 (14 Y)	Retinal atrophy, vascular attenuation, and bull’s eye maculopathy.	A central patch of increased AF at the macula surrounded by an annulus of decreased AF.	CRD	Severe disorganization of retinal lamination, with loss of EZ.	UD
26	20	20/70 and 20/60 (12 Y)	20/80 and 30/100 (20 Y)	Retinal atrophy, vascular attenuation, and macular pigment alteration.	Patch of increased AF at the macula.	CRD	Severe disorganization of retinal lamination with disrupted EZ.	NA
27A	30	HM OU (24 Y)	LP OU (28 Y)	Retinal atrophy, vascular attenuation, midperipheral bone spicules and macular atrophy.	Patches of decreased AF in the midperiphery with a patchy loss of AF at the macula.	Not classified	NA	NA
27B	18	LP OU (18 Y)	LP OU (18 Y)	Vascular attenuation, midperipheral bone spicules and macular atrophy.	Patches of decreased AF in the midperiphery with a patchy loss of AF at the macula.	Not classified	NA	NA
28A	28	20/300 OU (20 Y)	HM OU (28 Y)	vascular attenuation, midperipheral bone spicules and macular pigment alteration.	Patches of decreased AF in the midperiphery with a central patch of increased AF at the macula surrounded by an annulus of decreased AF.	CRD	Severe disorganization of retinal lamination with loss of EZ.	UD
28B	19	HM (6 Y)	HM OU (18 Y)	Coats’-like picture, retinal atrophy, vascular attenuation, scanty midperipheral bone spicules, macular atrophy and superior temporal peripheral cryotherapy scars.	Patches of decreased AF in the midperiphery with a patchy loss of AF at the macula.	CRD	Severe disorganization of retinal lamination with loss of EZ.	UD
28C	34	HM OU (29 Y)	LP OU (31 Y)	Vascular attenuation, midperipheral bone spicules, scanty nummular pigmentations, laser scars around the arcades and bull’s eye maculopathy.	Patches and nummular dots of decreased AF in the midperiphery with a central patch of increased AF at the macula surrounded by an annulus of decreased AF.	CRD	Partial disorganization of retinal lamination with loss of EZ.	NA
28D	30	LP OU (22 Y)	LP OU (30 Y)	Vascular attenuation, midperipheral bone spicules, scanty nummular pigmentations and macular atrophy, ARGUS II Implant OD.	Patches and nummular dots of decreased AF in the midperiphery and around the arcades with a patchy loss of AF at the macula.	CRD	Severe disorganization of retinal lamination with loss of EZ.	UD
29	17	20/160 OU (14 Y)	20/300 OU (15 Y)	Retinal atrophy, vascular attenuation, and macular atrophy.	Patches of decreased AF in the midperiphery with a central patch of increased AF at the macula surrounded by an annulus of decreased AF.	Not classified	NA	NA
30A	13	20/100 And 20/160 (8 Y)	20/100 20/160 (13 Y)	Retinal atrophy, vascular attenuation, scanty midperipheral bone spicules, and macular atrophy, left round pigmented lesion surrounded by lacunae (CHRPE).	Patches of decreased AF at the midperiphery and around the arcades with a central patch of increased AF at the macula surrounded by an annulus of decreased AF, left peripheral round lesion with decreased AF.	CRD.	Partial disorganization of retinal lamination with loss of EZ.	UD
30B	8	F and F (3 Y)	20/200 20/300 (8 Y)	Retinal atrophy, vascular attenuation, hypopigmented and macular pigment alteration.	A patch of decreased AF at the macula.	RCD	Unremarkable lamination with disrupted EZ.	UD
31	10	F and F (8 Y)	20/400 OU (10 Y)	Retinal atrophy, vascular attenuation, and macular pigment alteration.	A patch of decreased AF at the macula.	Generalized photoreceptor involvement.	Unremarkable lamination with disrupted EZ.	NA

## Data Availability

The original contributions presented in the study are included in the article.

## Supplementary Materials

The following supporting information can be downloaded at: https://www.mdpi.com/article/10.3390/genes15060762/s1, Figure S1: gene panels of the two clinically accredited laboratory centers, Figure S2: fundus images, Figure S3: fundus autofluorescence images, Figure S4: (a) OCT images, (b) fundus images; Figure S5: ERG data, Figure S6: pedigree of the probands.
