# Supplementary material for: The Clinical and Mutational Spectrum of Bardet–Biedl Syndrome in Saudi Arabia
Source: Genes (Basel). 2024 Jun 11;15(6):762. doi: 10.3390/genes15060762 (PMC11202873; doi:10.3390/genes15060762)
Supplement: Supplementary file 1 [file genes-15-00762-s001.zip › Figure S1 gene panels .pdf]

Retinal dystrophy panels of Bioscientia, Ingelheim, Germany includes: autosomal recessive retinitis pigmentosa (ARRP), BBS, cone-rod dystrophy (CRD), cone dystrophy (CD), and macular dystrophy (MD).

ARRP panel includes : *ABCA4, ABHD12, ACACB, ADGRA3, ADIPOR1, ABGL5, ARL2BP, ARL6, ASRGL1, BBS4, BEST1, C2ORF71, C8ORF37,CC2D2A, CDH16, CERKL, CLRN1, CNGA1, CNGB1, CRB1, CYP4V2, DHDDS, DHX38, DNAJC17, EMC1, EYS, FAM61A, FLVCR1, GNPTG, GNS, GRID2, HGSNAT, IDH3B, IFT140, IFT172, IMPG1, IMPG2, KIAA1549, KIZ, LAMA1, LRAT, MAK, MERTK, MFSD8, MPDZ, MTTP, MVK, NEK2, NEUROD1, NR2E3, NRL, PDE6A, PDE6B, PED6G, PLA2G5, PNPLA6, PRCD, PROM1, PFPR31, PRPH2, RBPH2, PBP3, PBP4, RDH11, RDH12, RDH5, RPGR, RHBDD2, RHO, RLBP1, RP1, RPE65, RPGRIP1, SAG, SLC7A14, SPATA7,TRNT1,TTC8, TPPA, TUB, TULP1,USH1C, USH2A, WDR19, ZNF513*

BBS panel includes: *BBS1-18, ALMS1*

CRD/DC/MD panels include: *ABCA4, ACBD5, ADAM9, AIPL1, BEST1, C1OTNF5, C21ORF2, C8ORF37, CABP4, CANCA1F, CANCA2D4, CDHR1, CERKL, CFH, CNGA3, CNGB3, CNNM4, CRX, DRAM2, ELOVL4, FBLN5, FSCN2, GUCA1A, GUCY2D, HMCN1, ITM2B, KCNV2, MERTK, MFSD8, MT-ATP6, MT-TL1, PCYT1A, PDE6C, PITPNM3, PLK4, POC1B, PRDM13, PROM1, PRPH2, RAB28, RAX2, RDH5, RGS9,RGS9BP, RIMS1, RP1L1, RPGR,RPGRPIP1, SEMA4A, TEAD1, TIMP3, TUBGGCP4, TUBGCP6, UNC119, WASF3*

Vision panel of King Faisal specialist hospital and Research center, Riyadh, Saudi Arabia includes: *ALMS1, CRB1, CHST6, CRYBA1, PRSS56, GUCY2D, SNRNP200, PDE6C, CNGA3, C8orf37, ABCA4, BBS10, CERKL, GPR125, NHS, LTBP2, GCNT2, IMPG2, CDHR1, GPR179, CRYGC, RLBP1, MIP, RP1L1, CHM, EYS, TULP1, IGFBP7, CYP1B1, LRAT, MERTK, CNNM4, RP1, RP2, LCA5, MFRP, CNGB1, CACNA1F, KCNV2, CRX, PROM1, TRPM1, PAX6, AIPL1, FAM161A, RPGRIP1, RAB3GAP2, RAB3GAP1, EFEMP1, BEST1, RPE65, EPHA2, FZD4, PRPH2, CRYAA, KCNJ13, NR2E3, BBS9, BBS1, BBS2, BBS5, BBS4, BBS7, SPATA7, CRYGD, NMNAT1, GALT, ARL6, LRP5, WDR19, SLC4A11, GDF3, SLC16A12, RGS9, RDH12, ADAM9.*

Figure S1: gene panels of the two clinically accredited laboratory centers
