# Supplementary material for: The Clinical and Mutational Spectrum of Bardet–Biedl Syndrome in Saudi Arabia
Source: Genes (Basel). 2024 Jun 11;15(6):762. doi: 10.3390/genes15060762 (PMC11202873; doi:10.3390/genes15060762)
Supplement: Supplementary file 1 [file genes-15-00762-s001.zip › Figure S2 Fundus images .pdf]

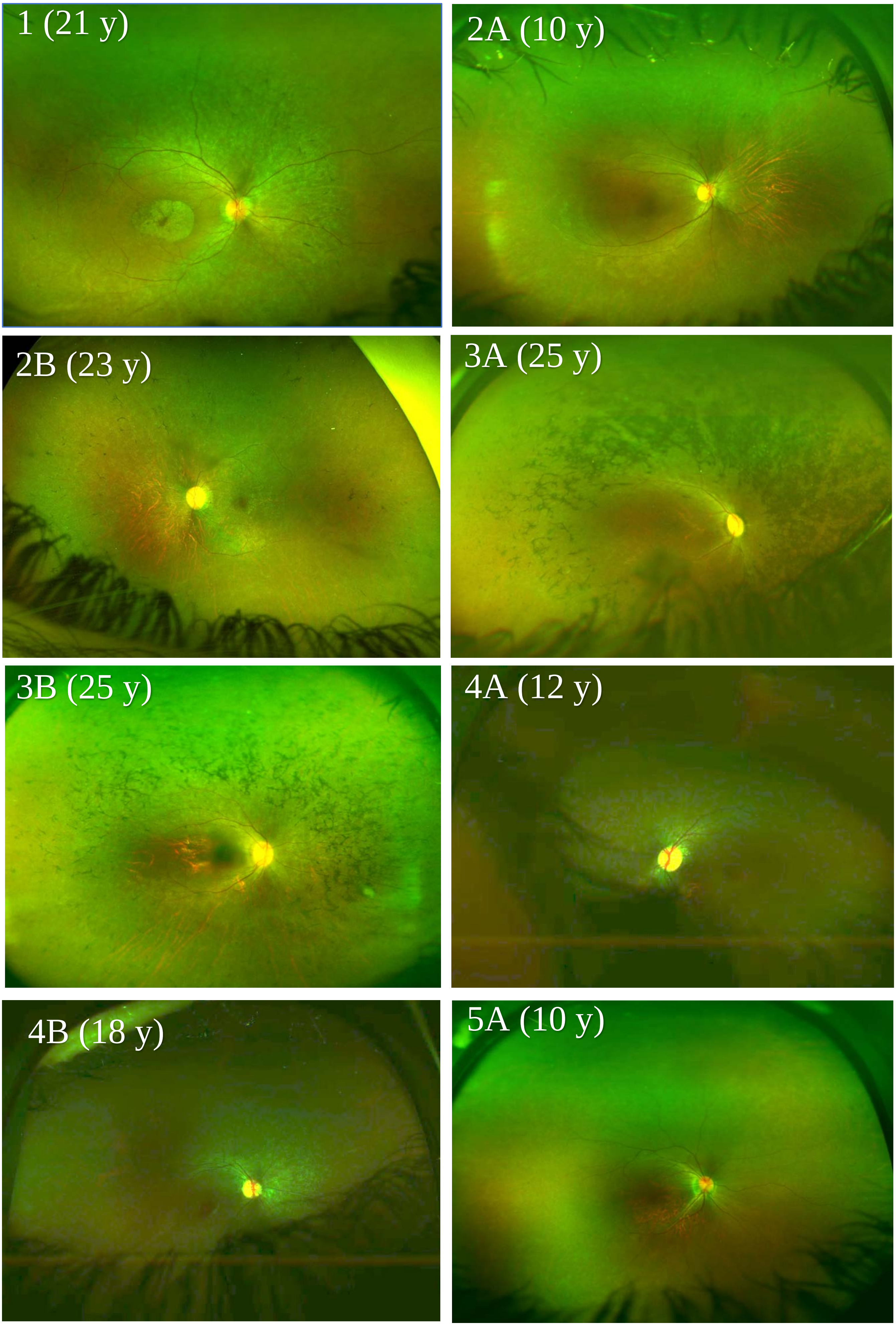
1 (21 y)
2A (10 y)
2B (23 y)
3A (25 y)
3B (25 y)
4A (12 y)
4B (18 y)
5A (10 y)

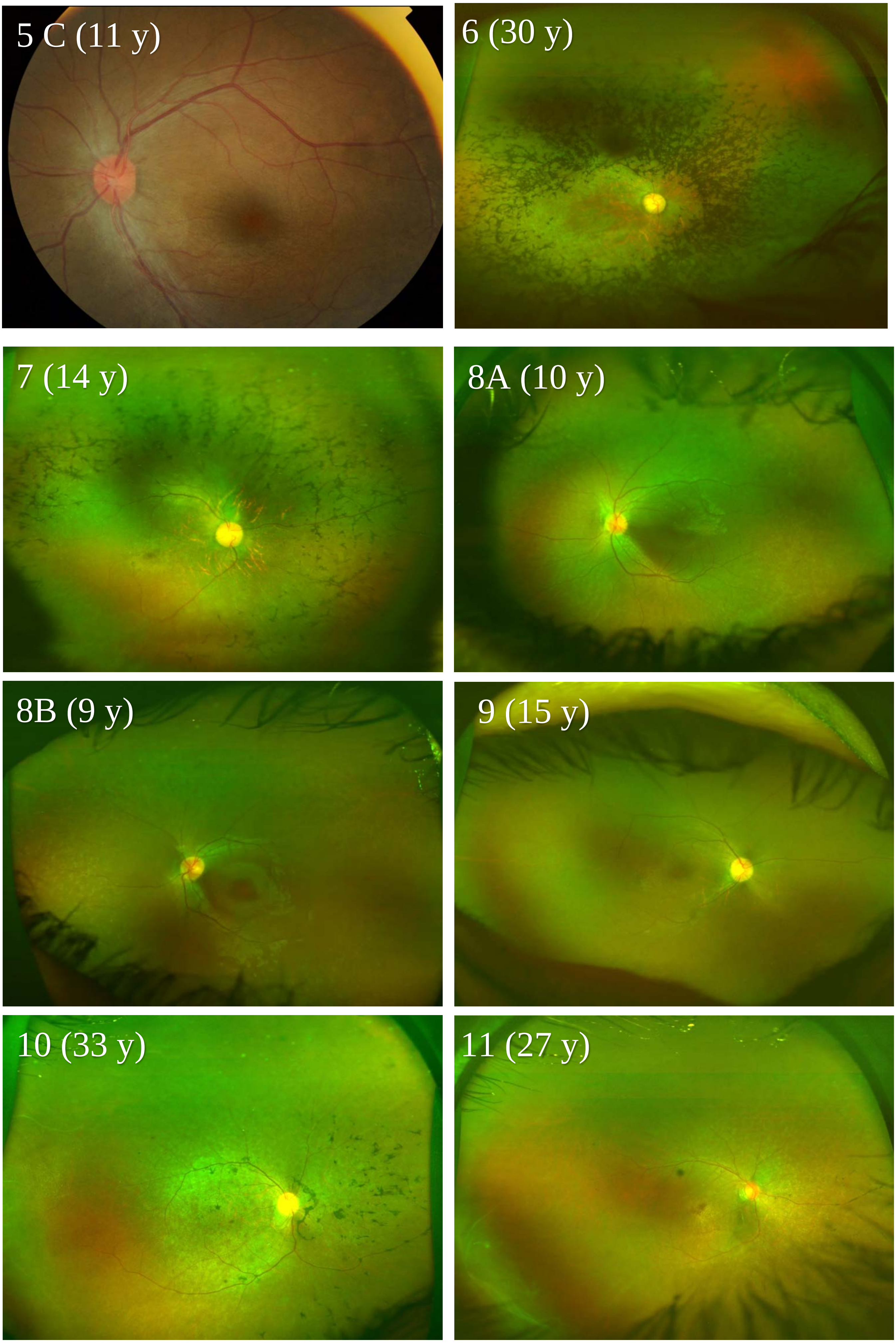
5 C (11 y)
6 (30 y)
7 (14 y)
8A (10 y)
8B (9 y)
9 (15 y)
10 (33 y)
11 (27 y)

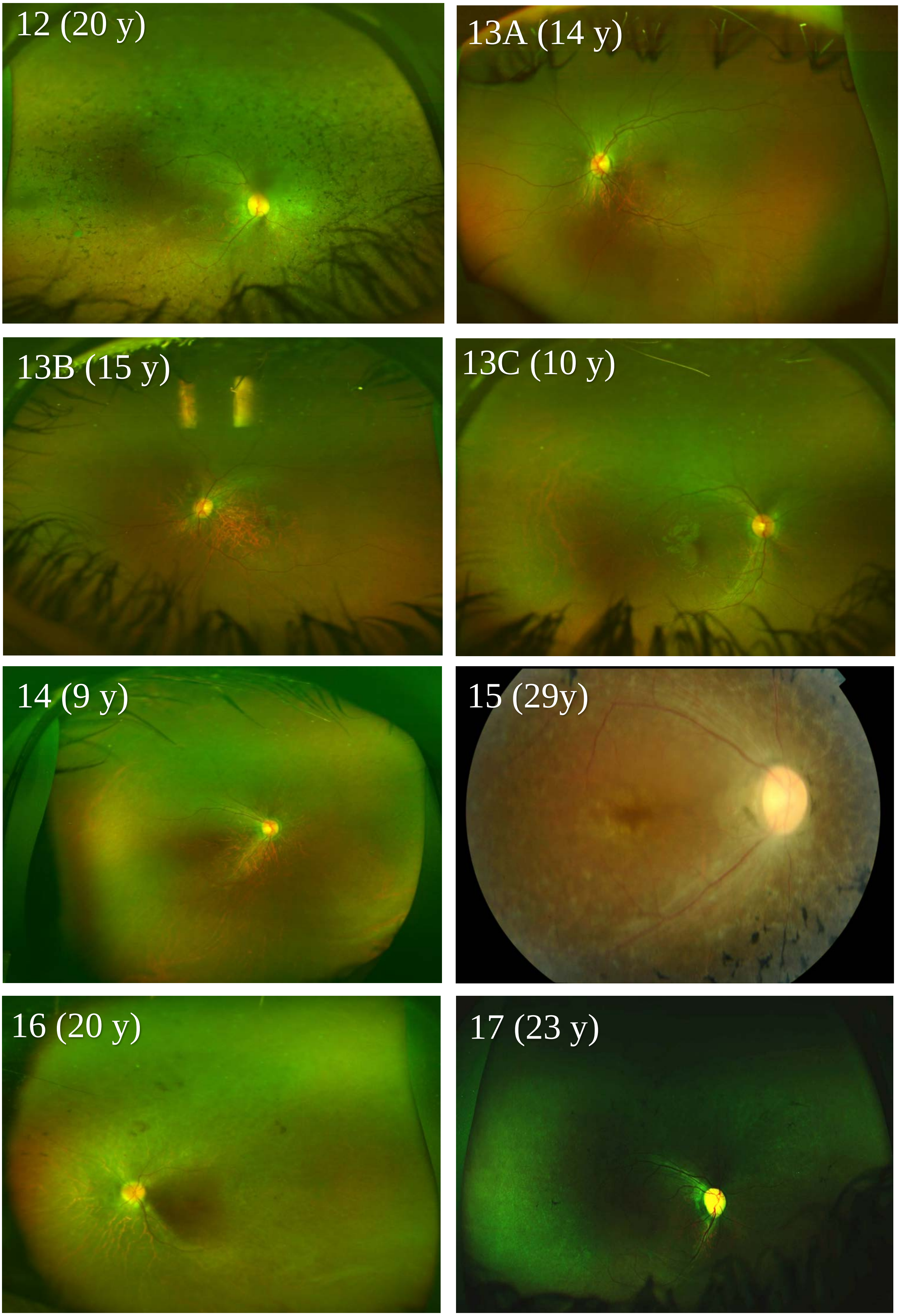
12 (20 y)
13A (14 y)
13B (15 y)
13C (10 y)
14 (9 y)
15 (29y)
16 (20 y)
17 (23 y)

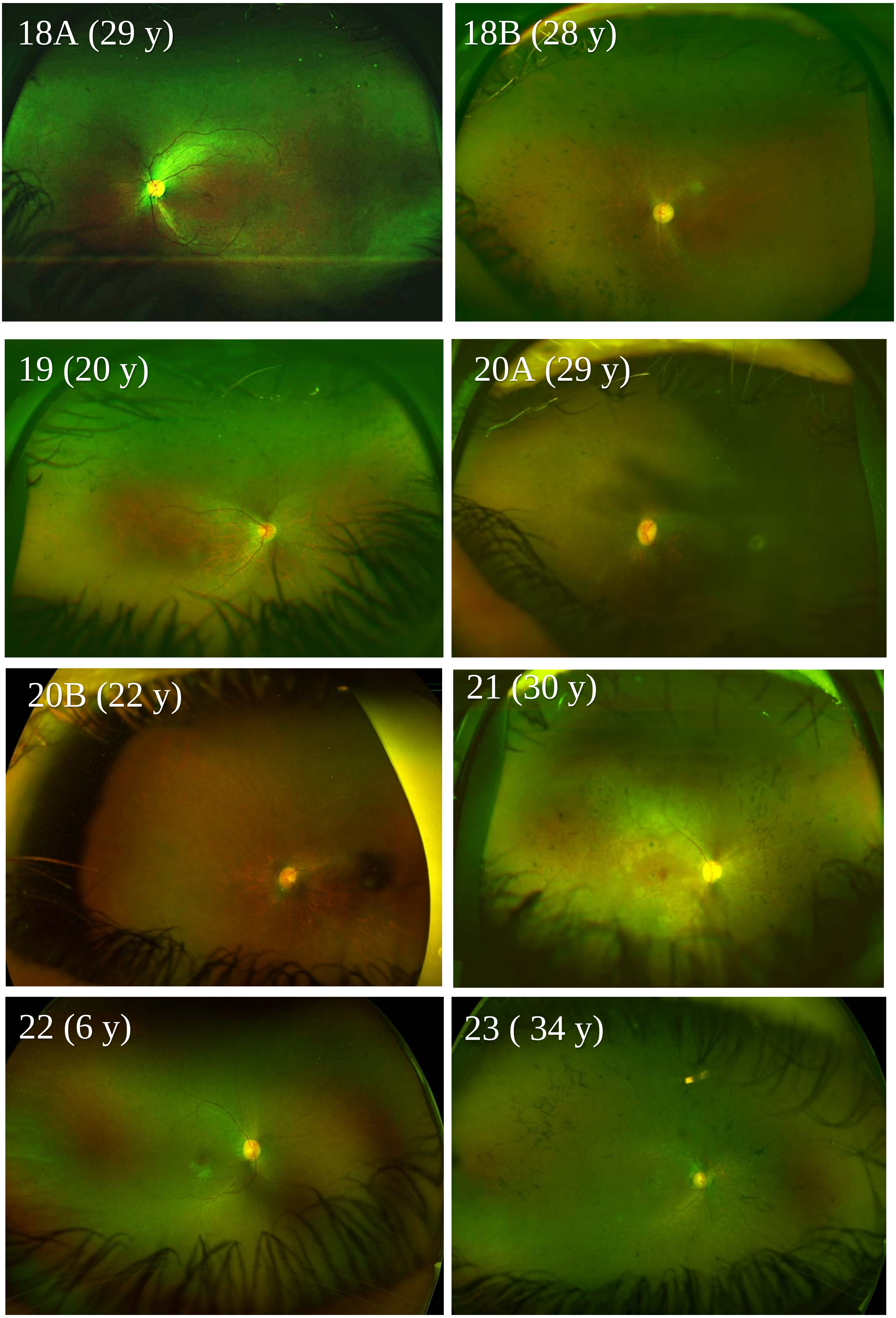
18A (29 y)
18B (28 y)
19 (20 y)
20A (29 y)
20B (22 y)
21 (30 y)
22 (6 y)
23 (34 y)

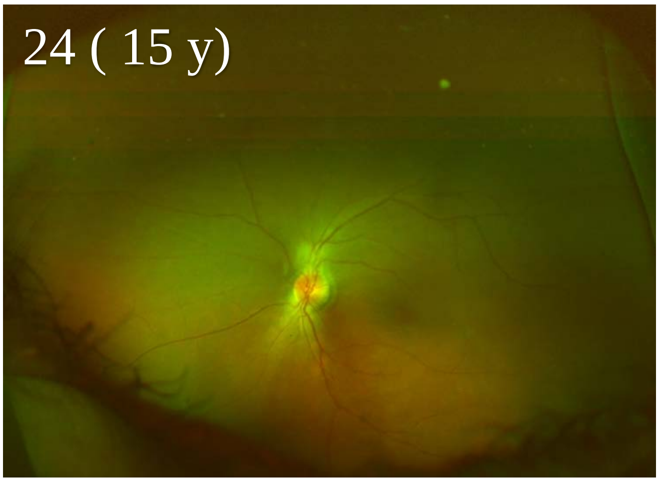
24 (15 y)
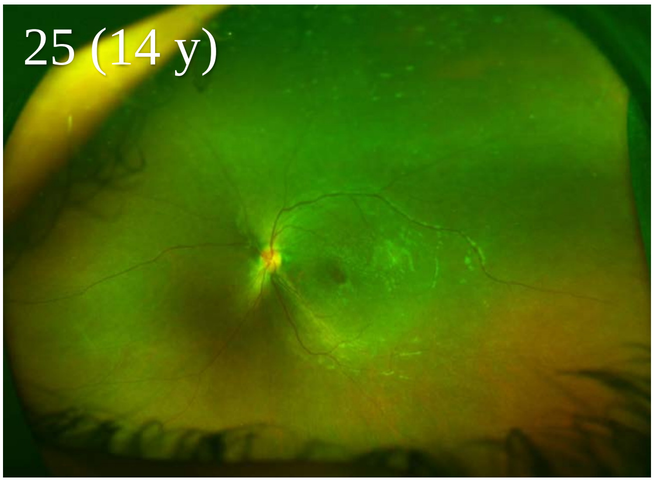
25 (14 y)
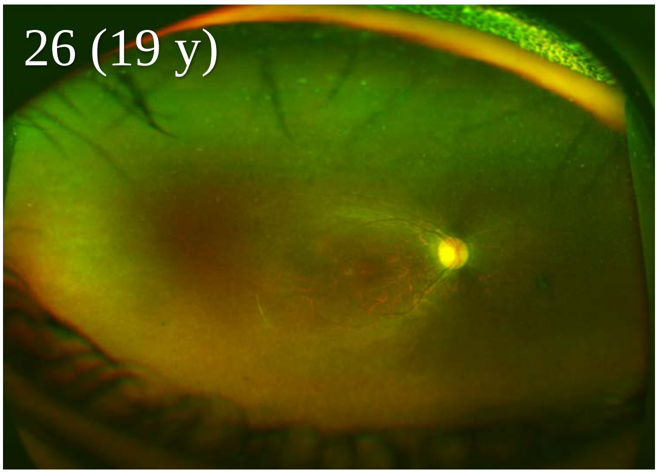
26 (19 y)
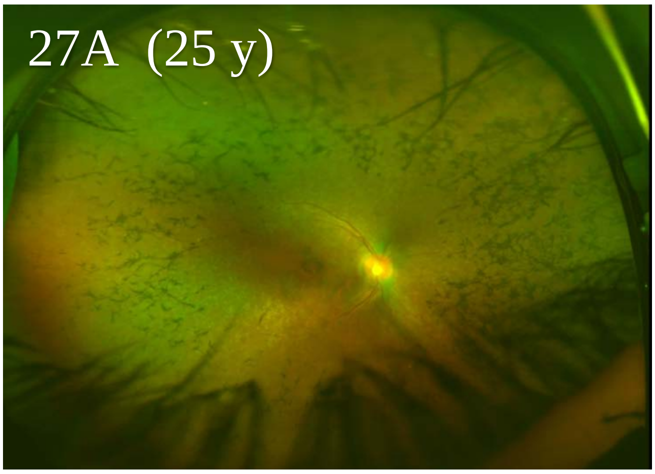
27A (25 y)
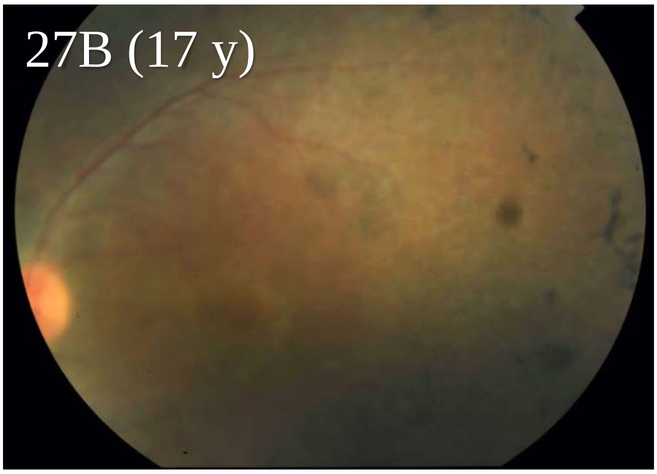
27B (17 y)
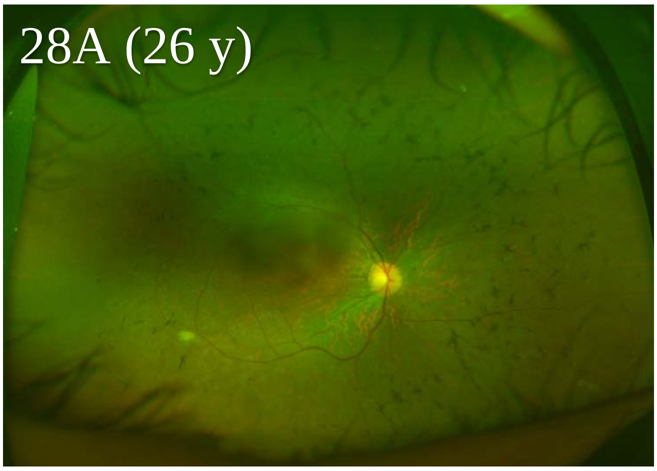
28A (26 y)
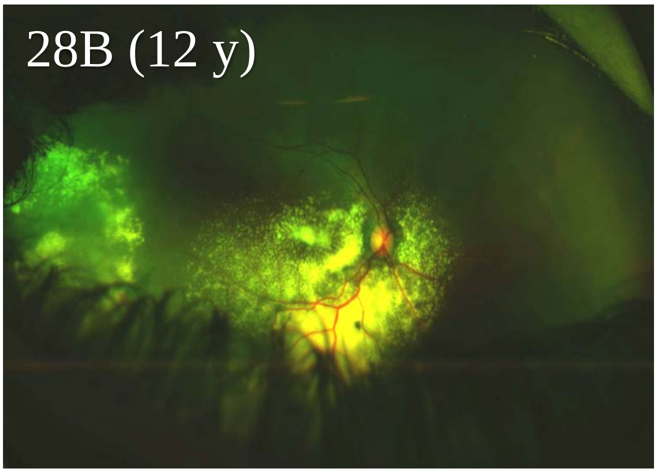
28B (12 y)
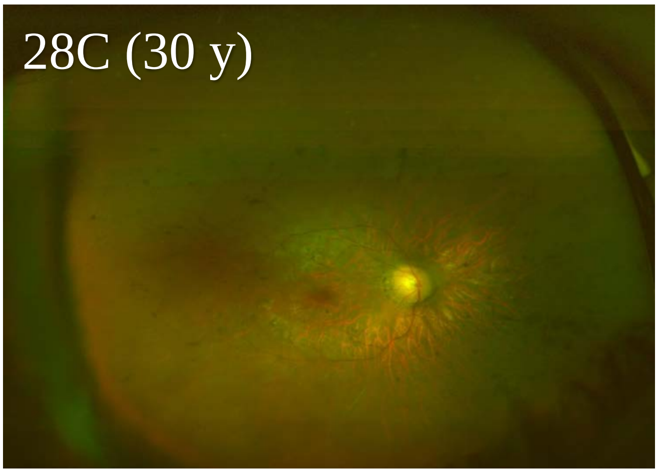
28C (30 y)

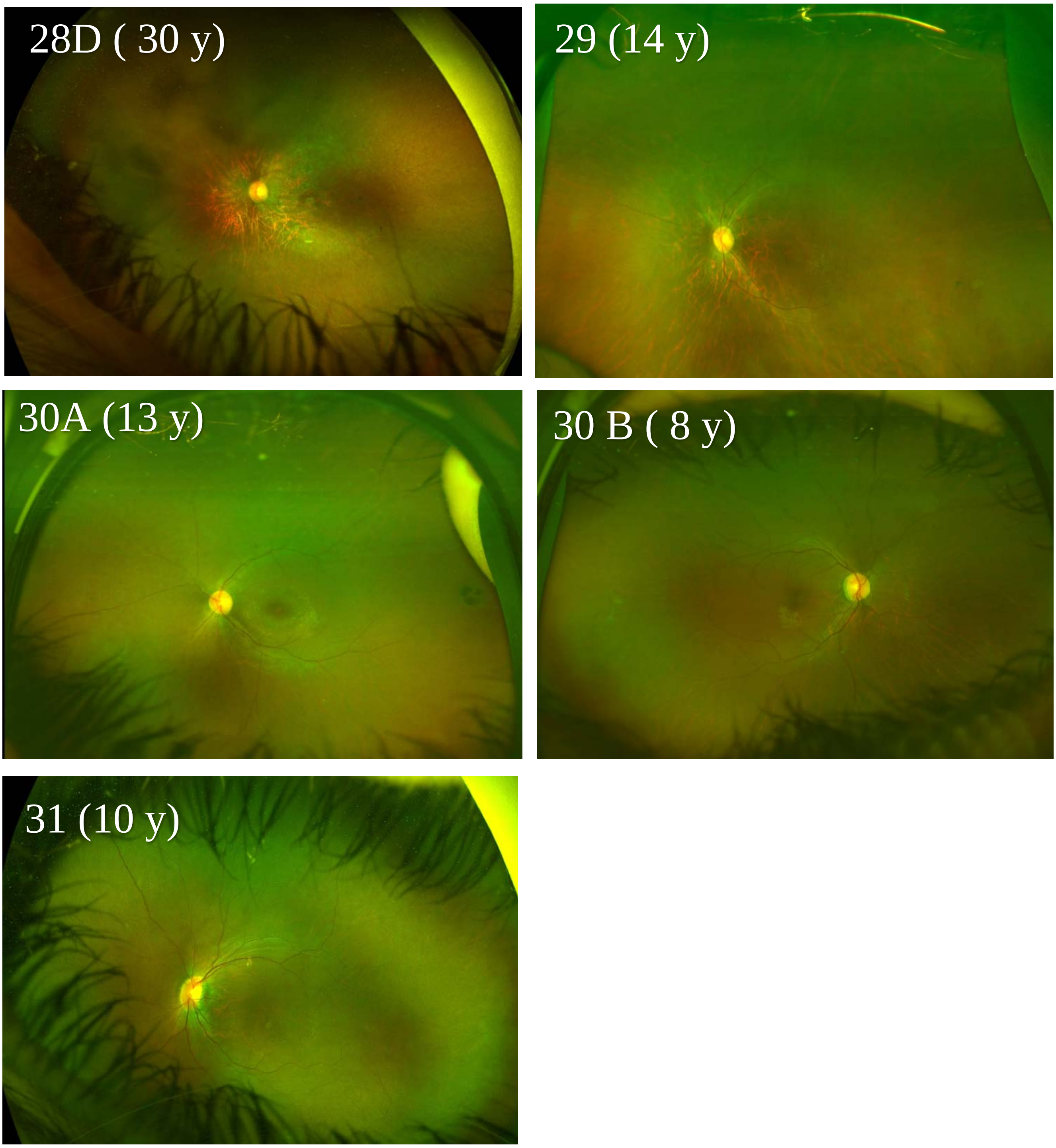

Figure S2 : Fundus images of the patients included in the study. Retinal images of patient *5B* were not available; the number between brackets represents the age of the patient at the time of imaging.
