# Supplementary material for: The Clinical and Mutational Spectrum of Bardet–Biedl Syndrome in Saudi Arabia
Source: Genes (Basel). 2024 Jun 11;15(6):762. doi: 10.3390/genes15060762 (PMC11202873; doi:10.3390/genes15060762)
Supplement: Supplementary file 1 [file genes-15-00762-s001.zip › Figure S3 FAF images .pdf]

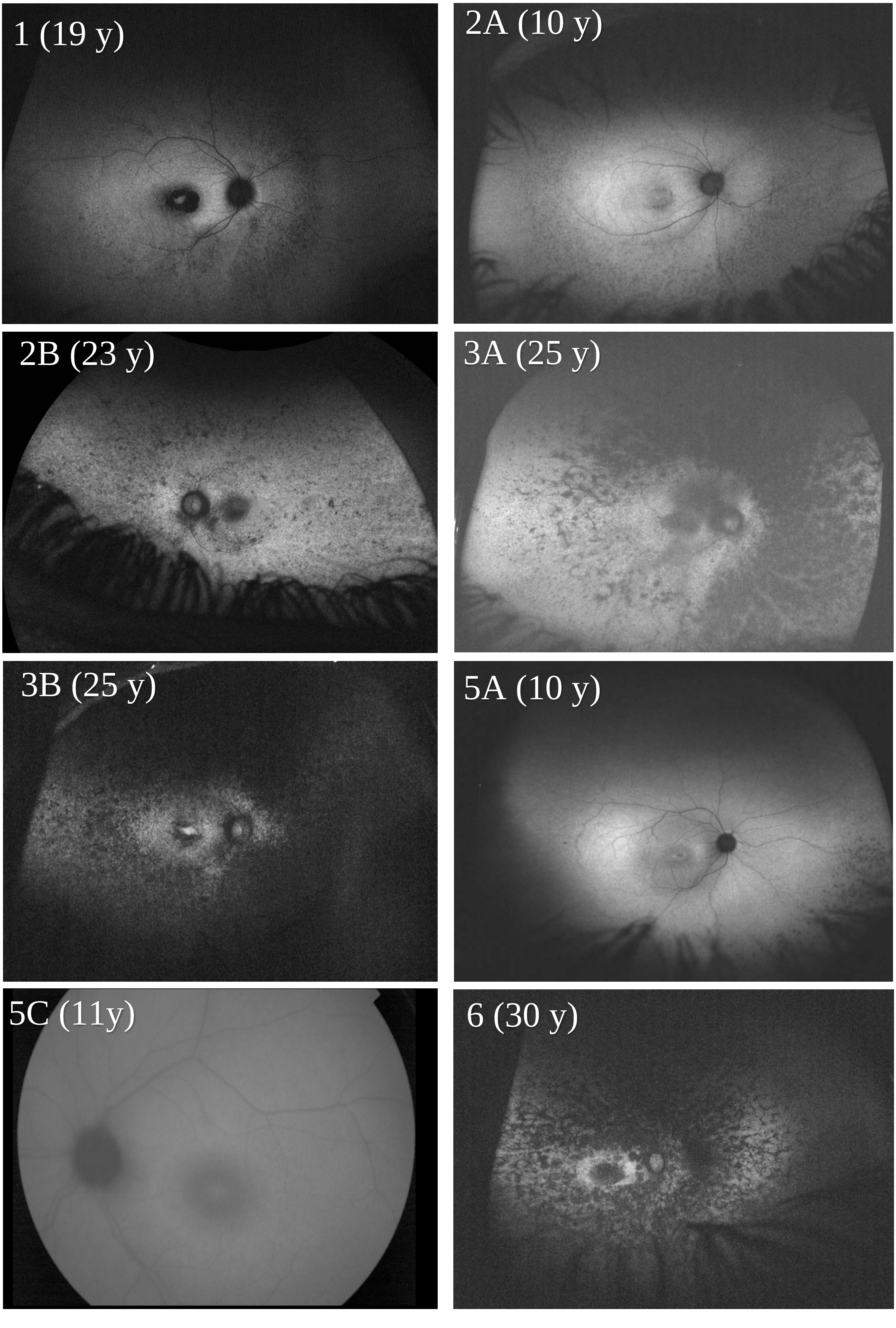
1 (19 y)
2A (10 y)
2B (23 y)
3A (25 y)
3B (25 y)
5A (10 y)
5C (11y)
6 (30 y)

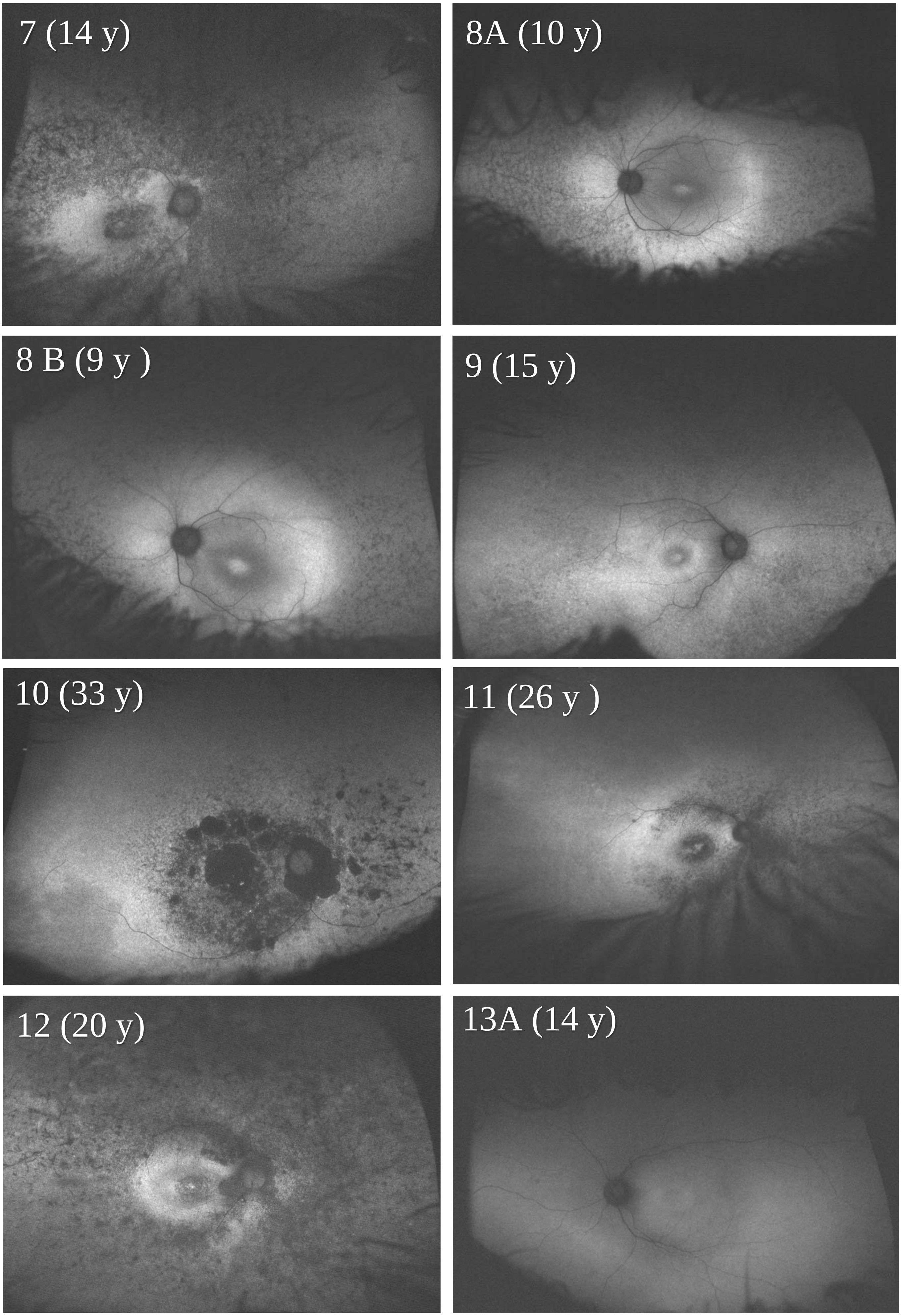
7 (14 y)
8A (10 y)
8 B (9 y)
9 (15 y)
10 (33 y)
11 (26 y)
12 (20 y)
13A (14 y)

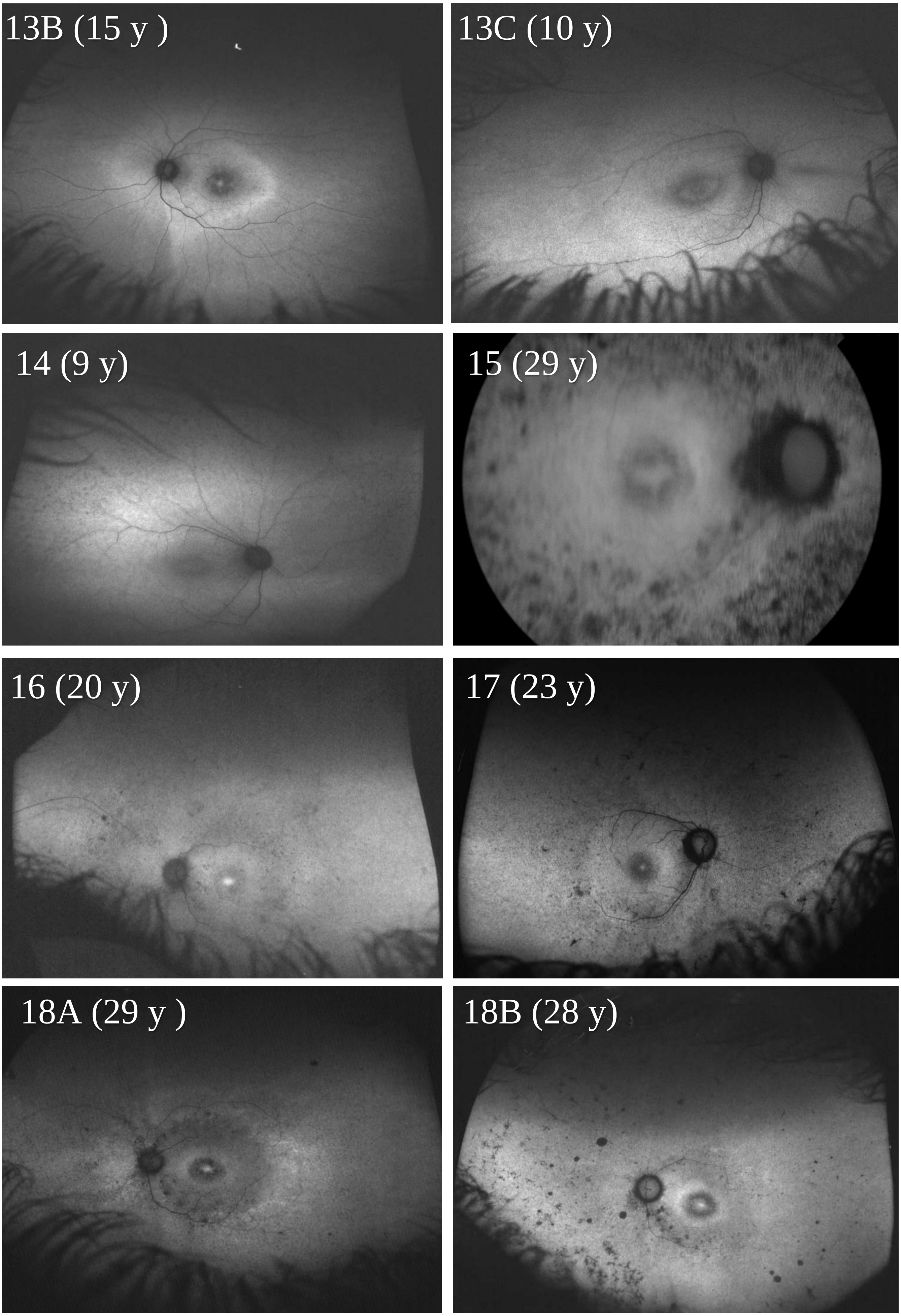
13B (15 y)
13C (10 y)
14 (9 y)
15 (29 y)
16 (20 y)
17 (23 y)
18A (29 y)
18B (28 y)

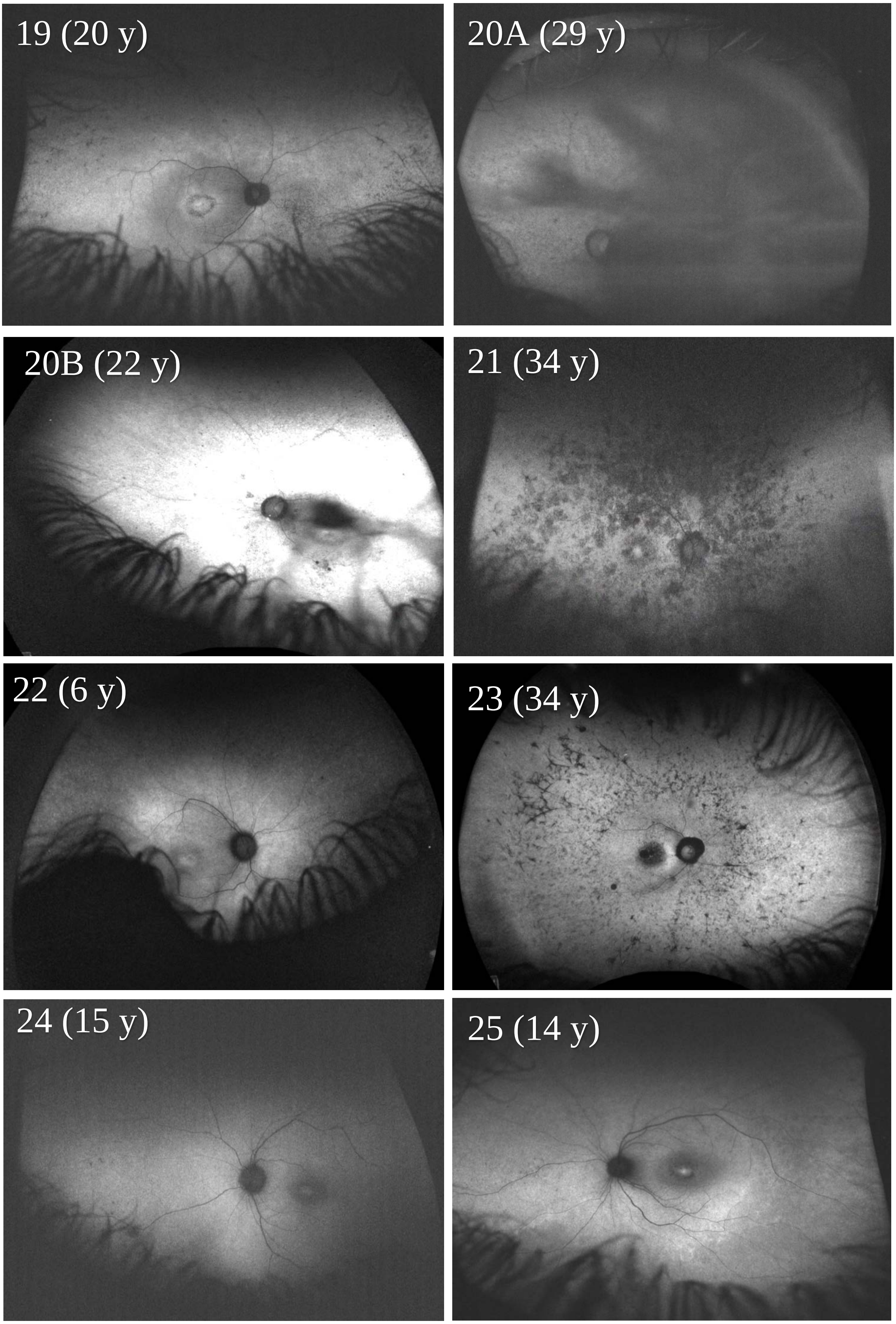
19 (20 y)
20A (29 y)
20B (22 y)
21 (34 y)
22 (6 y)
23 (34 y)
24 (15 y)
25 (14 y)

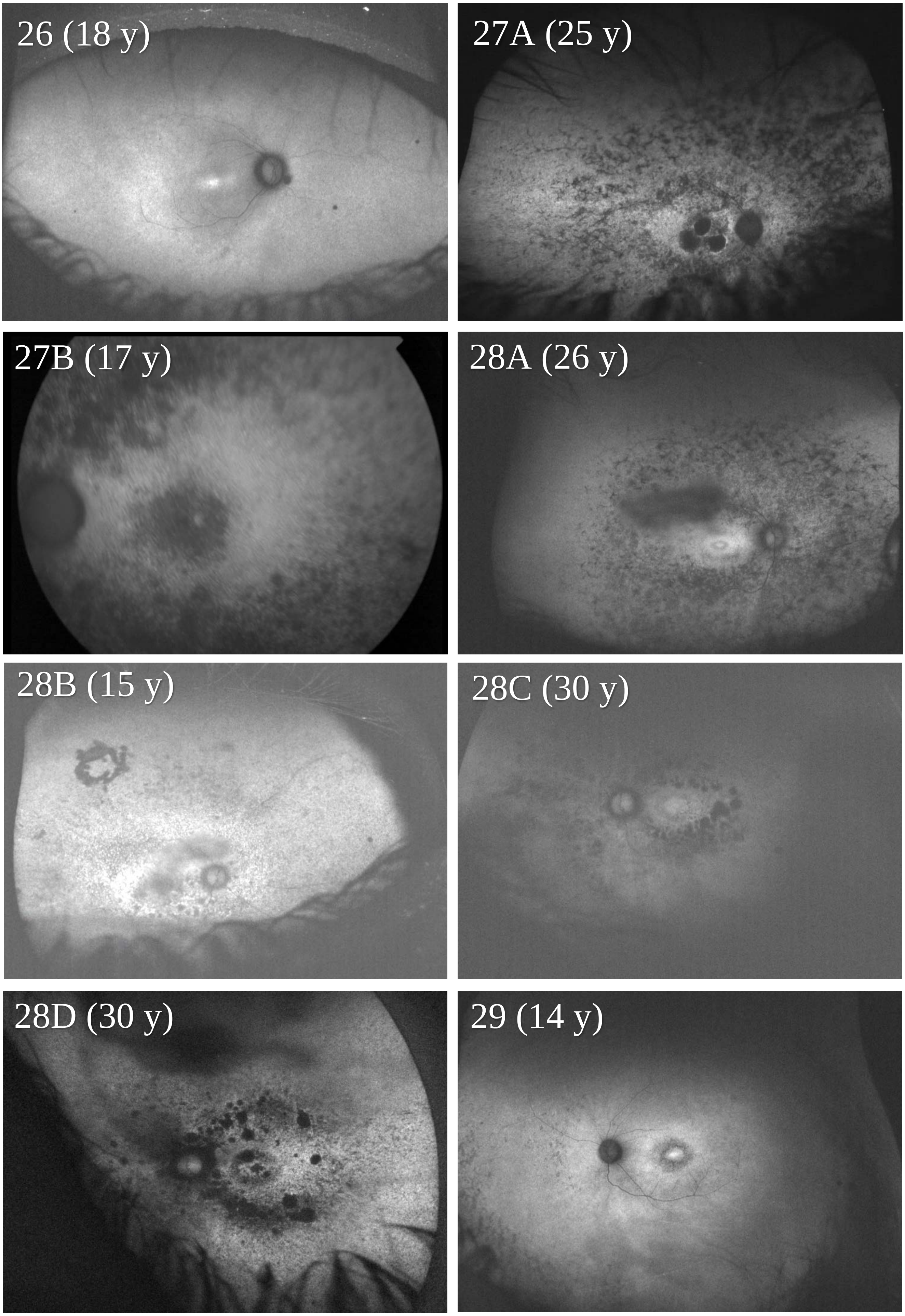
26 (18 y)
27A (25 y)
27B (17 y)
28A (26 y)
28B (15 y)
28C (30 y)
28D (30 y)
29 (14 y)

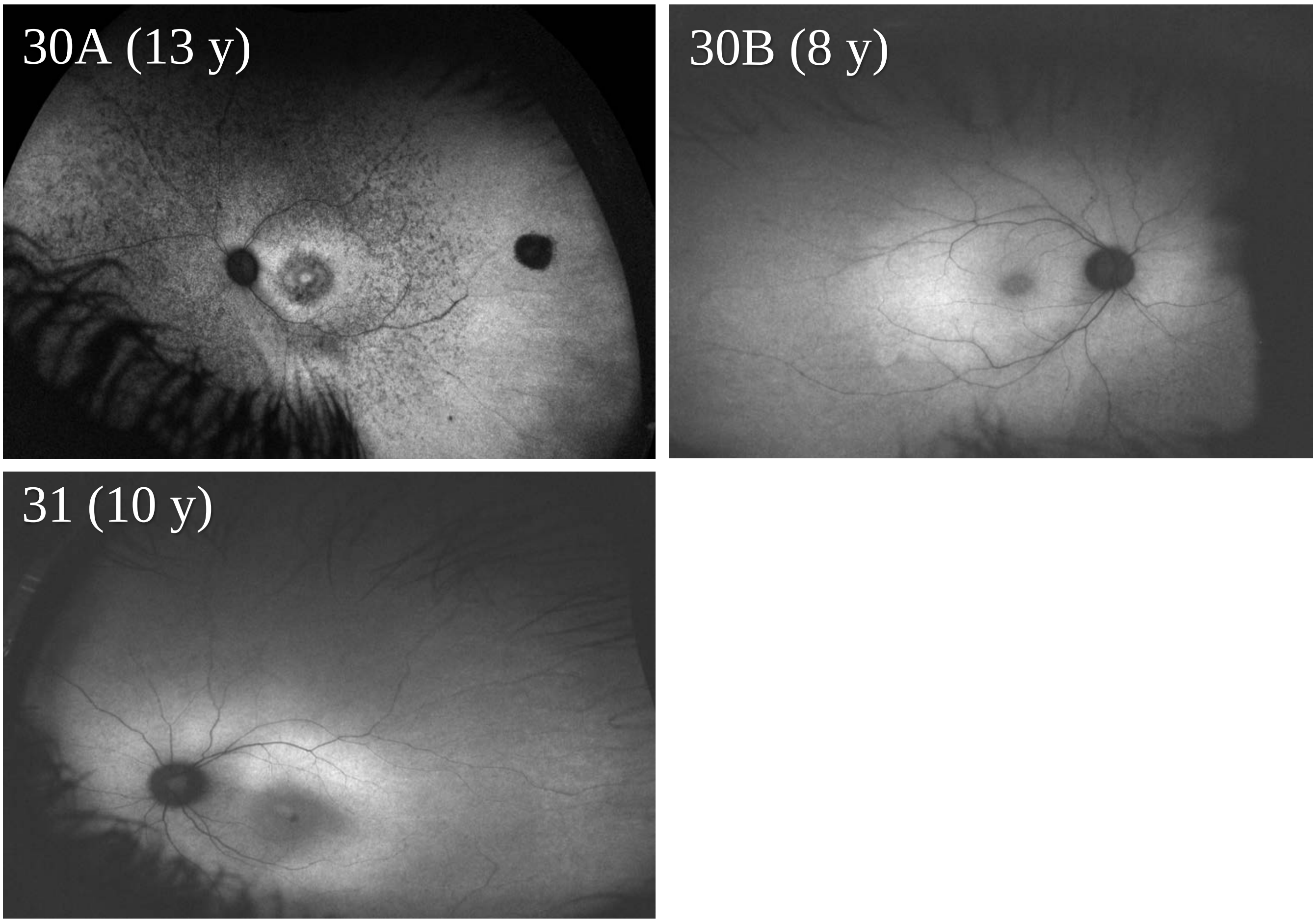

Figure S3 : Fundus autofluorescence images of most patients. Description of the autofluorescence patterns is given in Table 2 and summarized in the manuscript; the number between parenthesis represents the age of the patient at the time of imaging; images of patients *4A,4B* and *5B* were not available.
