# Supplementary material for: The Clinical and Mutational Spectrum of Bardet–Biedl Syndrome in Saudi Arabia
Source: Genes (Basel). 2024 Jun 11;15(6):762. doi: 10.3390/genes15060762 (PMC11202873; doi:10.3390/genes15060762)
Supplement: Supplementary file 1 [file genes-15-00762-s001.zip › Figure S4.pdf]

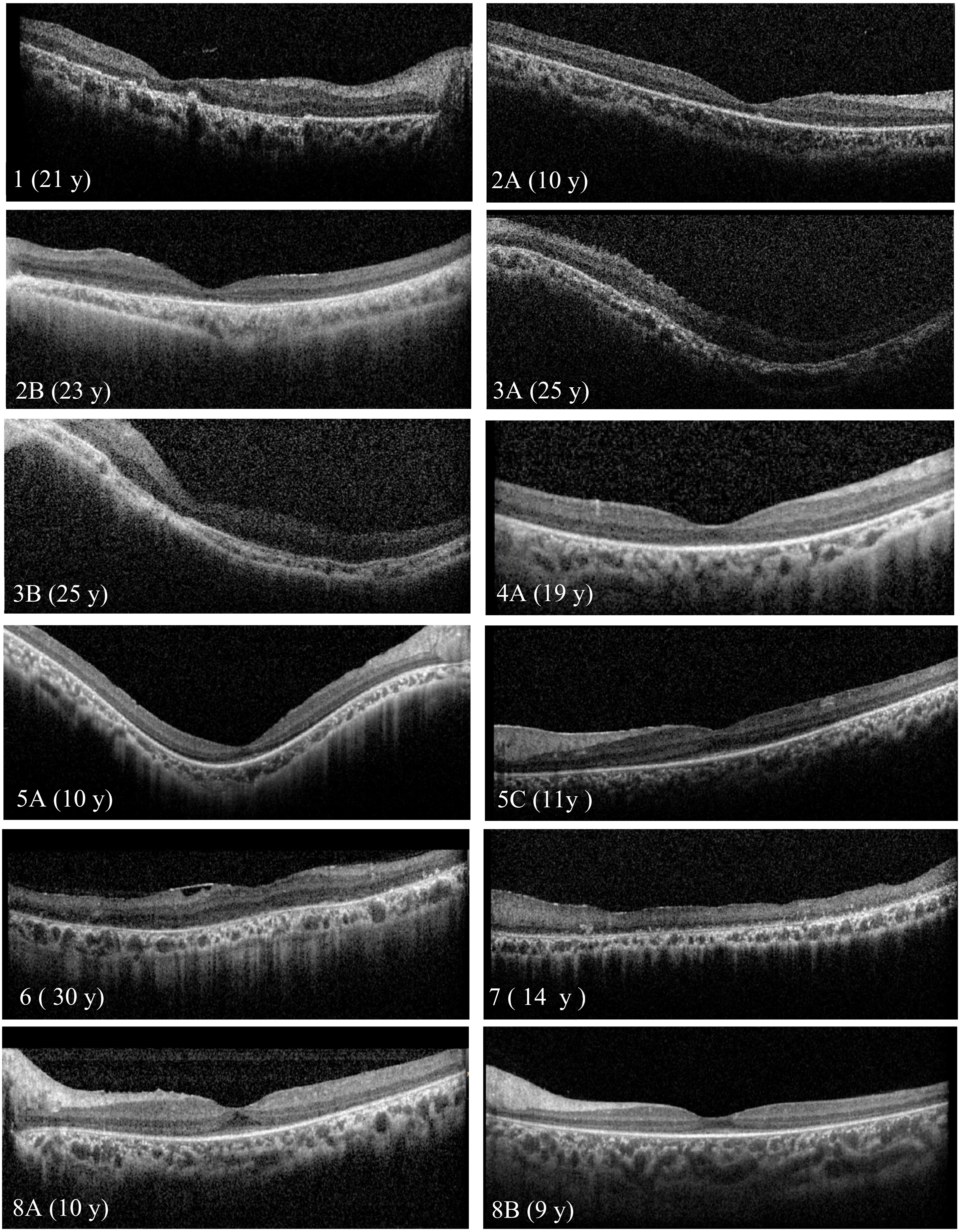
1 (21 y)
2A (10 y)
2B (23 y)
3A (25 y)
3B (25 y)
4A (19 y)
5A (10 y)
5C (11y)
6 (30 y)
7 (14 y)
8A (10 y)
8B (9 y)

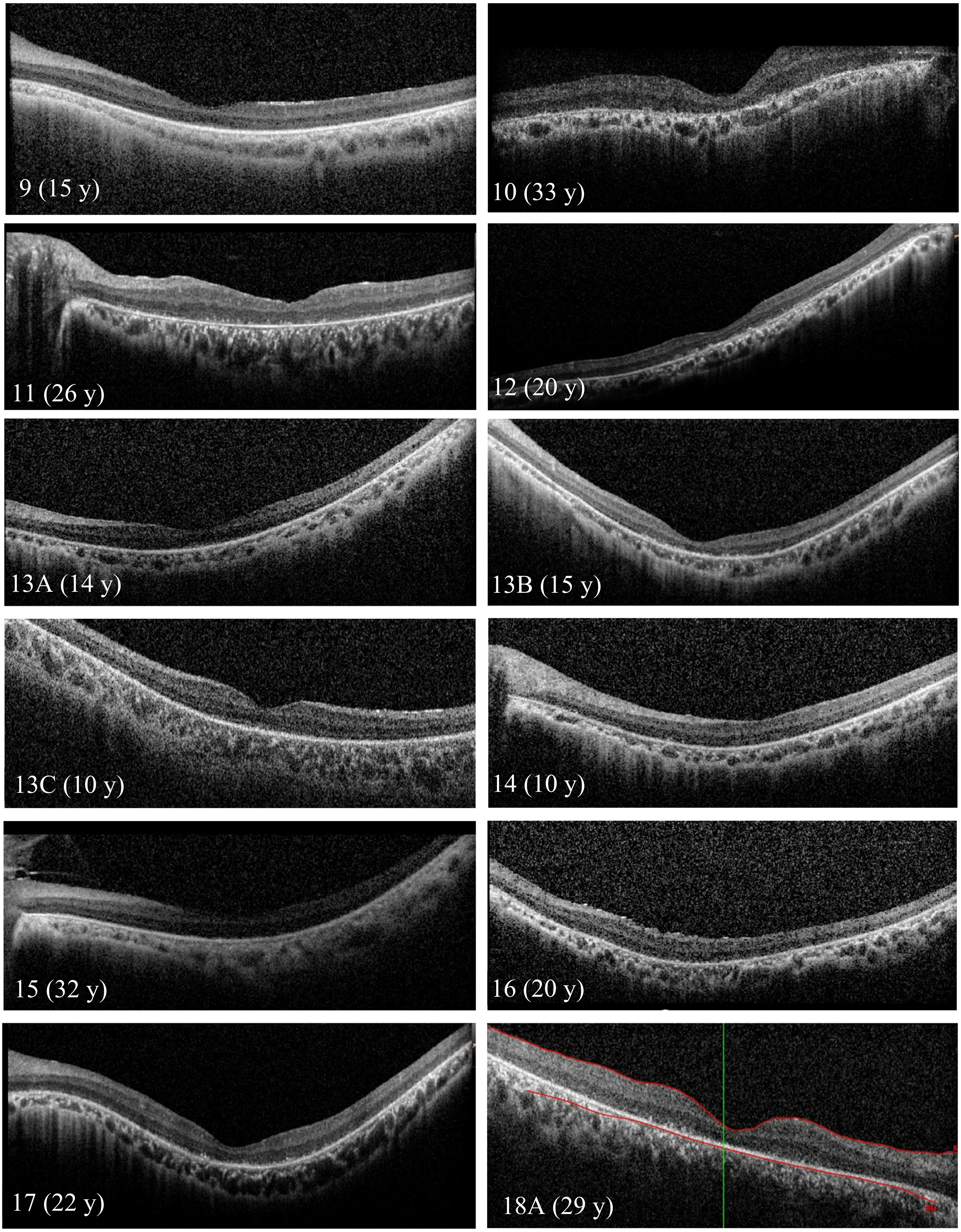
9 (15 y)
10 (33 y)
11 (26 y)
12 (20 y)
13A (14 y)
13B (15 y)
13C (10 y)
14 (10 y)
15 (32 y)
16 (20 y)
17 (22 y)
18A (29 y)

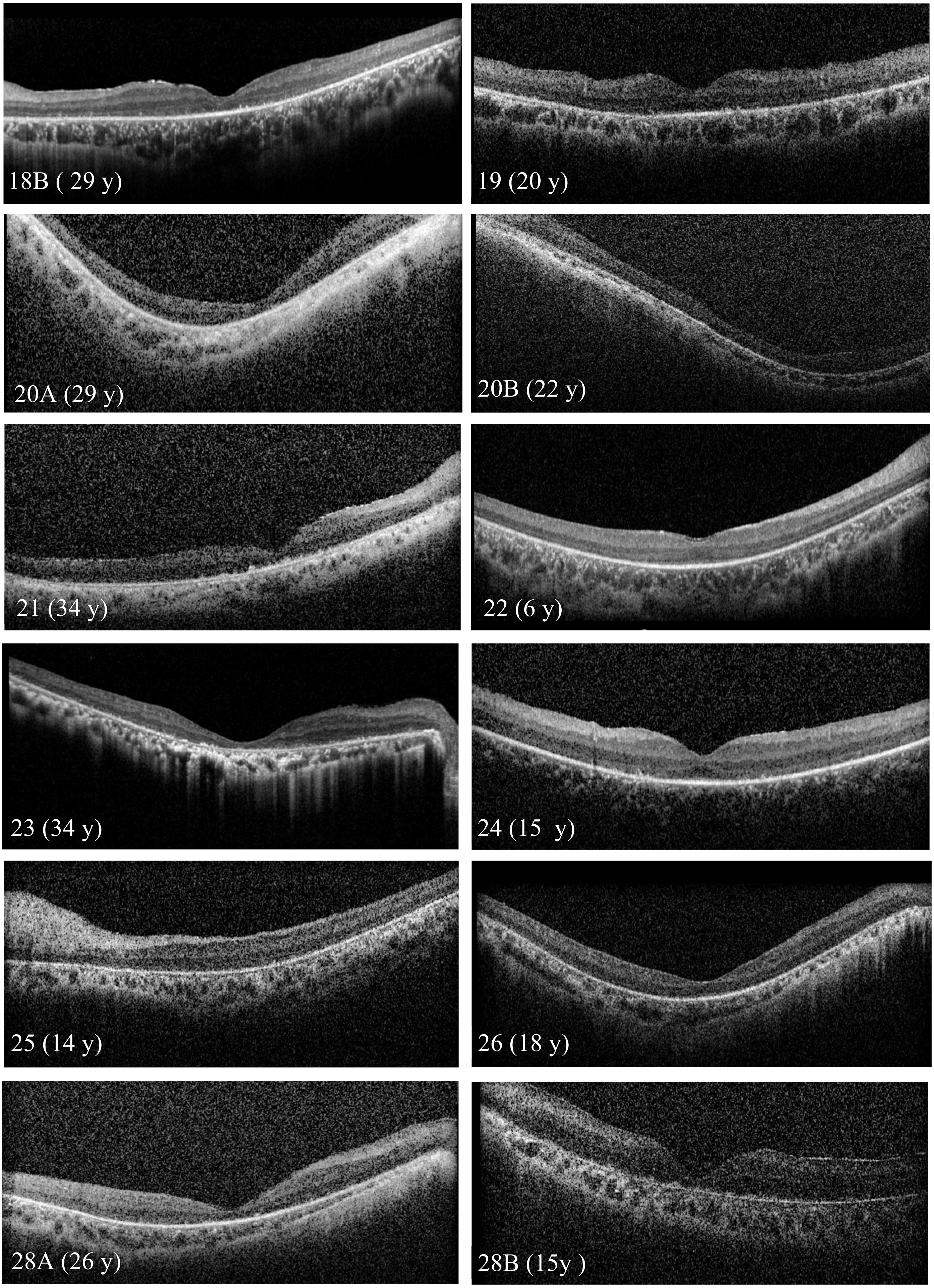
18B (29 y)
19 (20 y)
20A (29 y)
20B (22 y)
21 (34 y)
22 (6 y)
23 (34 y)
24 (15 y)
25 (14 y)
26 (18 y)
28A (26 y)
28B (15y)

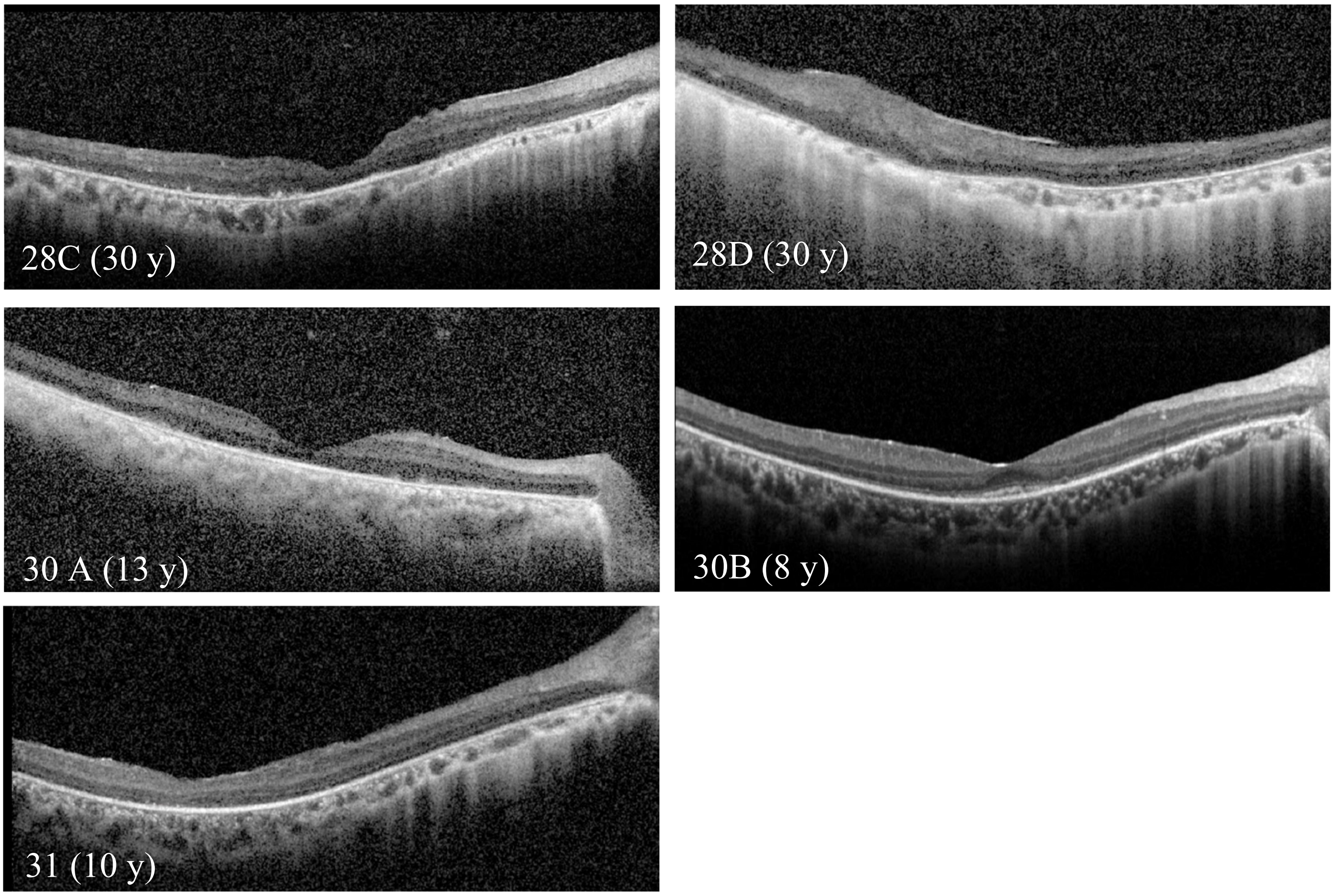
28C (30 y)
28D (30 y)
30 A (13 y)
30B (8 y)
31 (10 y)

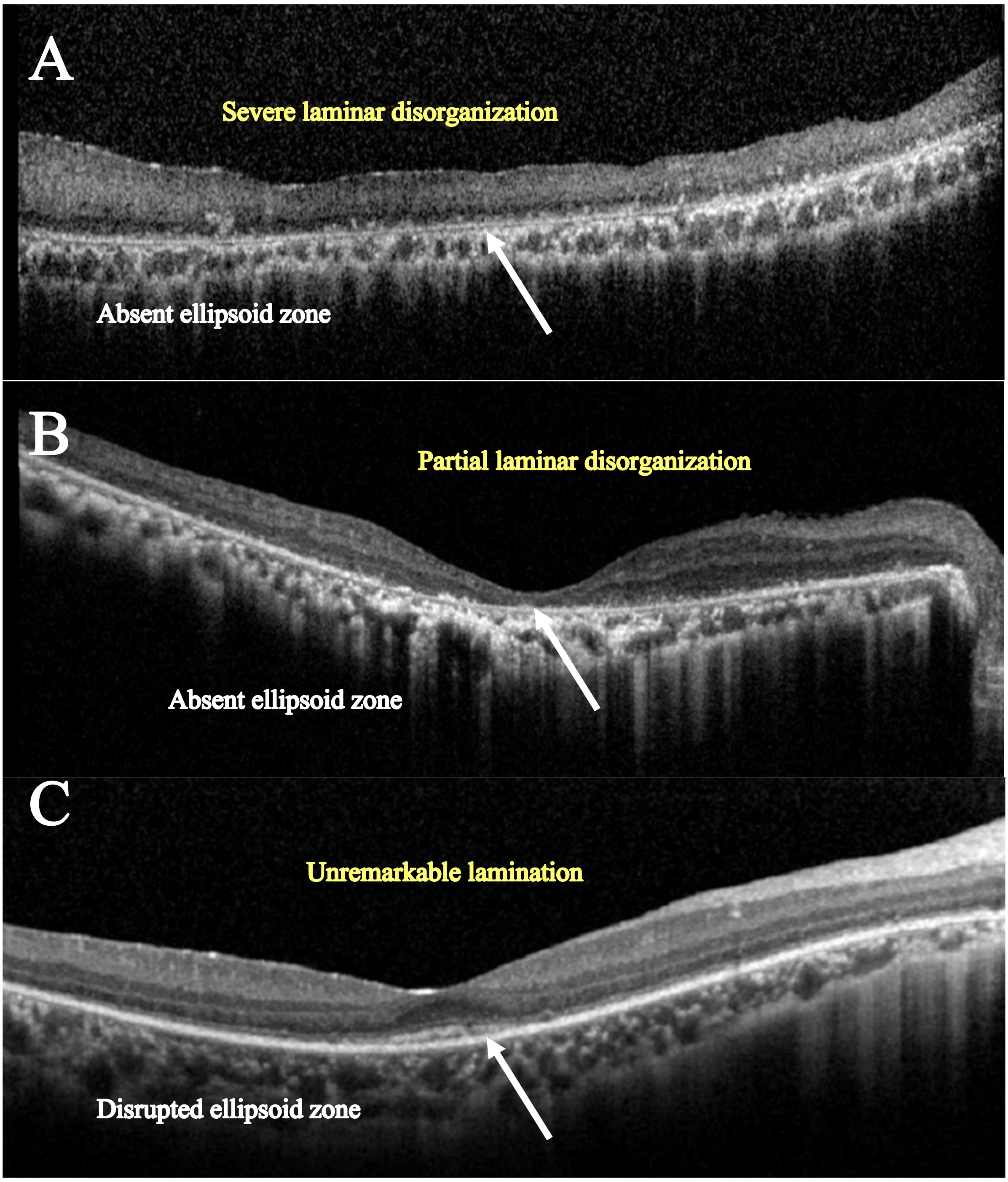

Figure S4 a: Optical coherence tomography scans of patient in this series; scans of patients 4B, 5B 27A, 27B and 29 were not available. The number between parenthesis represents the age of the patient at the time of scan. b Examples of the three patterns of retinal lamination A: severe laminar disorganization B: partial laminar disorganization C: unremarkable lamination. The white arrows demonstrate the state of the ellipsoid zone (EZ) which was categorized as either absent or disrupted.
