# Supplementary material for: The Clinical and Mutational Spectrum of Bardet–Biedl Syndrome in Saudi Arabia
Source: Genes (Basel). 2024 Jun 11;15(6):762. doi: 10.3390/genes15060762 (PMC11202873; doi:10.3390/genes15060762)
Supplement: Supplementary file 1 [file genes-15-00762-s001.zip › Figure S5 ERG data.pdf]

A

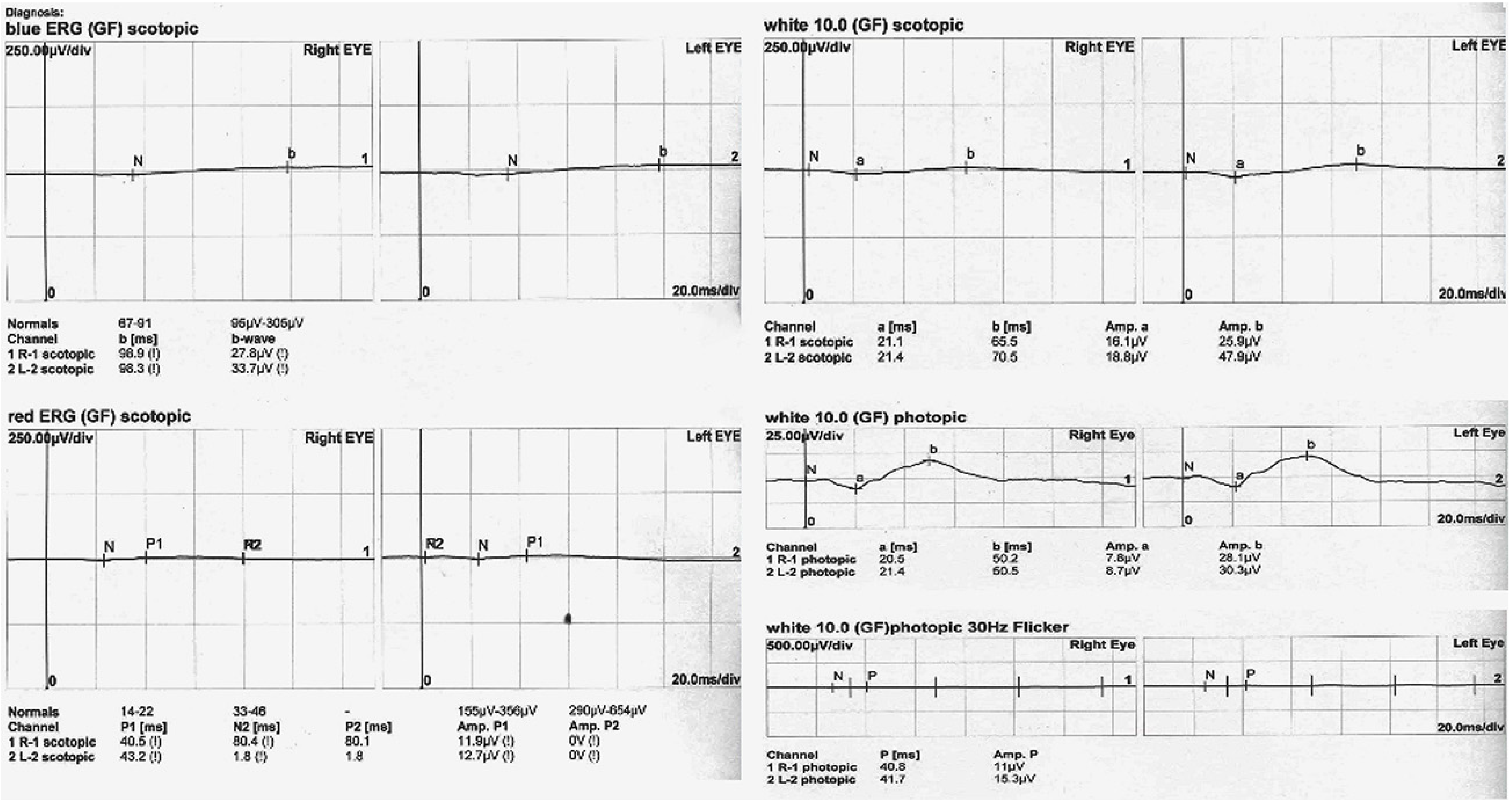
Diagnosis:
blue ERG (GF) scotopic
250.00µV/div
Right EYE
Left EYE
20.0ms/div
Normals 67-91 95µV-305µV
Channel b [ms] b-wave
1 R-1 scotopic 98.9 (!) 27.8µV (!)
2 L-2 scotopic 98.3 (!) 33.7µV (!)
red ERG (GF) scotopic
250.00µV/div
Right EYE
Left EYE
20.0ms/div
Normals 14-22 33-46 - 155µV-356µV 290µV-654µV
Channel P1 [ms] N2 [ms] P2 [ms] Amp. P1 Amp. P2
1 R-1 scotopic 40.5 (!) 80.4 (!) 80.1 11.9µV (!) 0V (!)
2 L-2 scotopic 43.2 (!) 1.8 (!) 1.8 12.7µV (!) 0V (!)
white 10.0 (GF) scotopic
250.00µV/div
Right EYE
Left EYE
20.0ms/div
Channel a [ms] b [ms] Amp. a Amp. b
1 R-1 scotopic 21.1 65.5 16.1µV 25.9µV
2 L-2 scotopic 21.4 70.5 18.8µV 47.9µV
white 10.0 (GF) photopic
25.00µV/div
Right Eye
Left Eye
20.0ms/div
Channel a [ms] b [ms] Amp. a Amp. b
1 R-1 photopic 20.5 50.2 7.8µV 28.1µV
2 L-2 photopic 21.4 50.5 8.7µV 30.3µV
white 10.0 (GF)photopic 30Hz Flicker
500.00µV/div
Right Eye
Left Eye
20.0ms/div
Channel P [ms] Amp. P
1 R-1 photopic 40.8 11µV
2 L-2 photopic 41.7 15.3µV

B

Diagnosis:

blue ERG (GF) scotopic

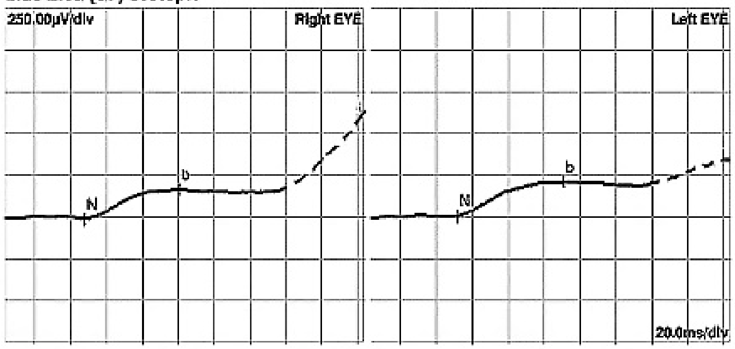

Normals	67-91	95µV-305µV
Channel	b [ms]	b-wave
1 R-1 scotopic		
9 R-1 scotopic	80.6	177.3µV
5 R-1 scotopic		
2 L-2 scotopic		
4 L-2 scotopic		
6 L-2 scotopic	91.6 (!)	205.6µV

red ERG (GF) scotopic

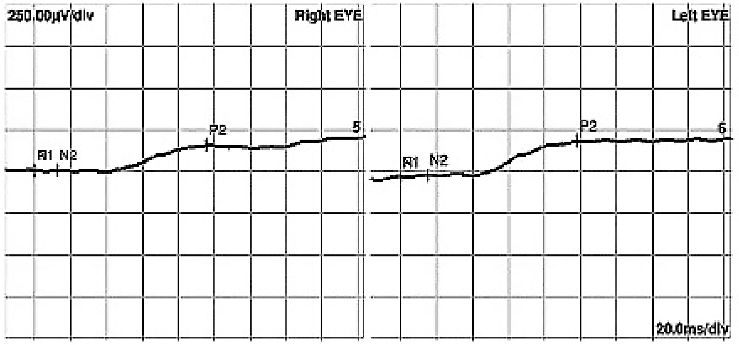

Normals	14-22	33-46	-	155µV-356µV	290µV-654µV
Channel	P1 [ms]	N2 [ms]	P2 [ms]	Amp. P1	Amp. P2
1 R-1 scotopic					
9 R-1 scotopic					
5 R-1 scotopic	0.0 (!)	12.9 (!)	96.8	0V (!)	153.6µV (!)
2 L-2 scotopic					
4 L-2 scotopic					
6 L-2 scotopic	0.0 (!)	15.8 (!)	96.8	0V (!)	202.1µV (!)

white 10.0 (GF) scotopic

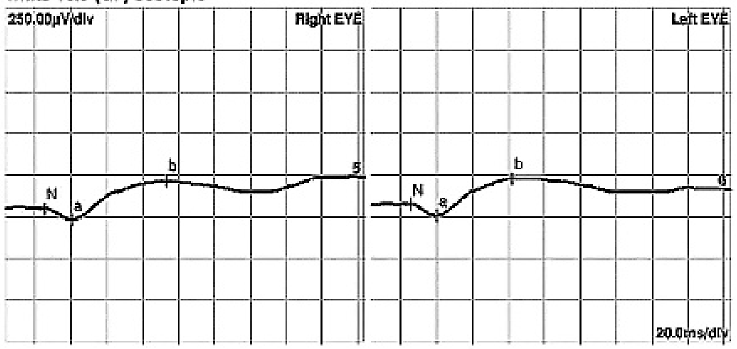

Channel	a [ms]	b [ms]	Amp. a	Amp. b
1 R-1 scotopic				
9 R-1 scotopic				
5 R-1 scotopic	21.1	79.6	64.2µV	231.1µV
2 L-2 scotopic				
4 L-2 scotopic				
6 L-2 scotopic	20.4	62.6	68.9µV	228.8µV

white 10.0 (GF) photopic

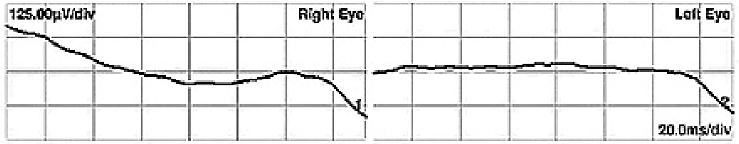

Channel	a [ms]	b [ms]	Amp. a	Amp. b
1 R-1 photopic	0.0	0.0		
2 L-2 photopic	0.0	0.0		

white 10.0 (GF)photopic 30Hz Flicker

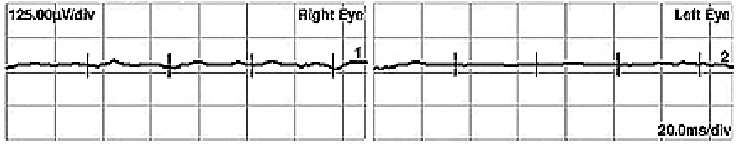

Channel	P [ms]	Amp. P
1 R-1 photopic	0.0	
2 L-2 photopic	0.0	

Figure S5 : Electroretinographic (ERG) recordings from the two patients with detectable ERGs in this study. ***A:*** ERGs recorded from patient 5B at the age of 12 months under chloral hydrate sedation using Burian-Allen electrodes. The scotopic responses were recorded after 20 minutes of dark adaptation. The scotopic responses to a blue flash showed severe amplitude reduction; the response to red flash under dark adaptation showed no discernable response. The dark-adapted bright flash response showed severe reduction of the a- and b-wave amplitudes. The printing scale precluded commenting on the waveform. The light-adapted single flash response showed amplitude reduction to approximately 25% of normal with severe b-wave delay*B:* ERG recordings from patient 13B at the age of 15 years. The dark-adapted (DA) response to a blue flash is moderately reduced. Note the absence of the x-wave component, which originate from the dark-adapted cones, of the DA response to a red flash with relative preservation of the b-wave. The DA response to a bright flash showed a subnormal a-wave amplitude with moderate reduction of the b-wave amplitude. The light adapted responses were undetectable.
