# Supplementary figures and images for: The Clinical and Mutational Spectrum of Bardet–Biedl Syndrome in Saudi Arabia
Source: Genes (Basel). 2024 Jun 11;15(6):762. doi: 10.3390/genes15060762 (PMC11202873; doi:10.3390/genes15060762)

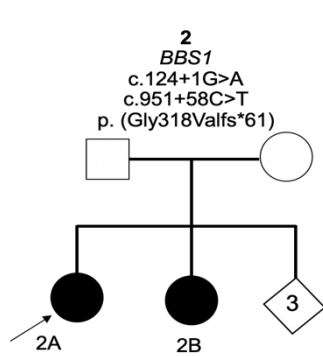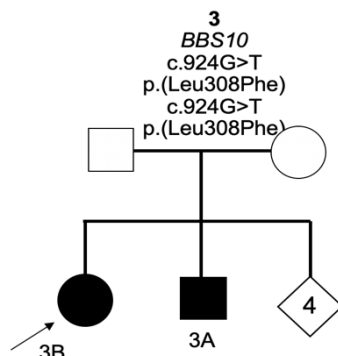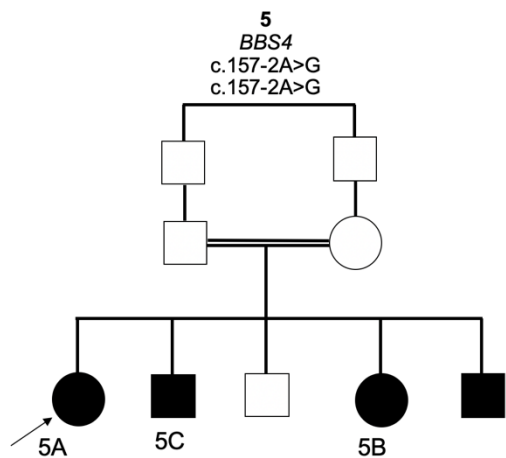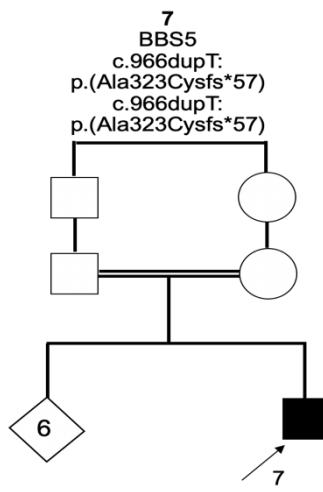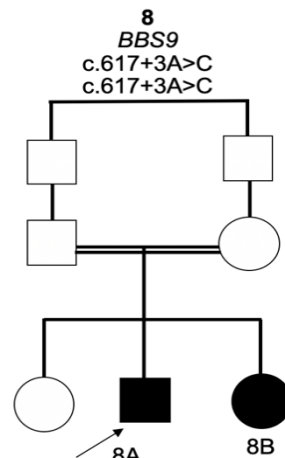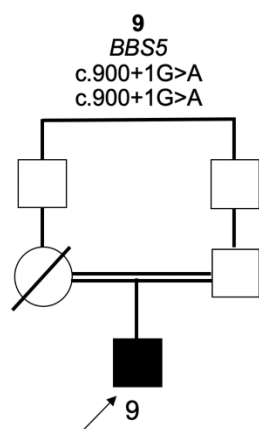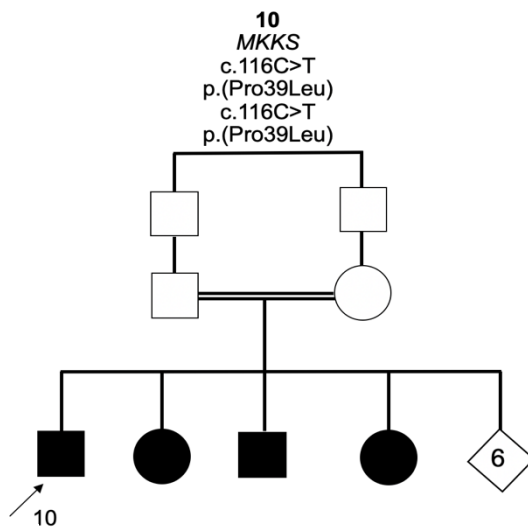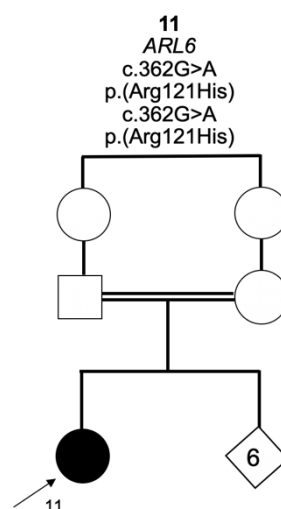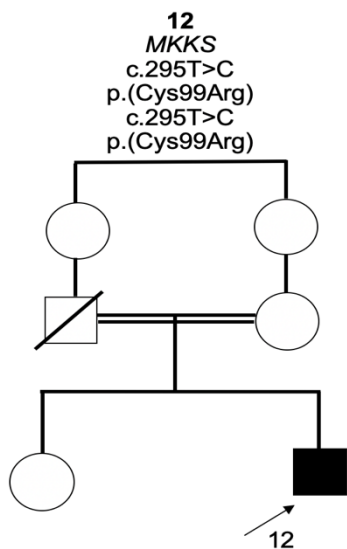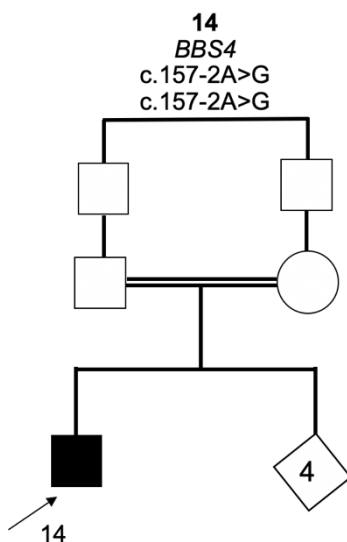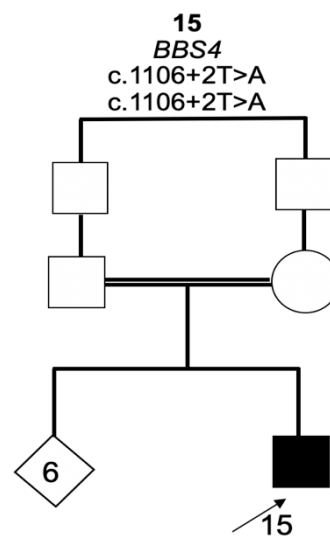

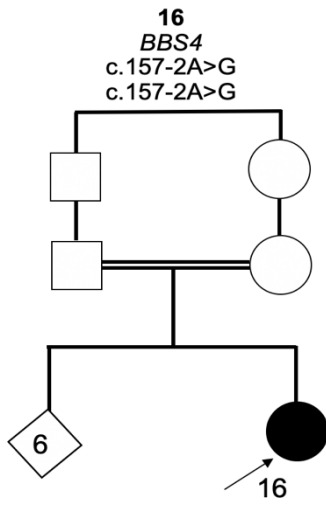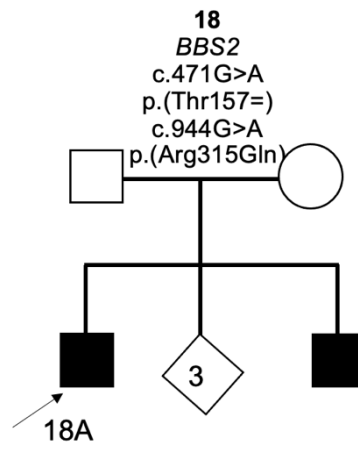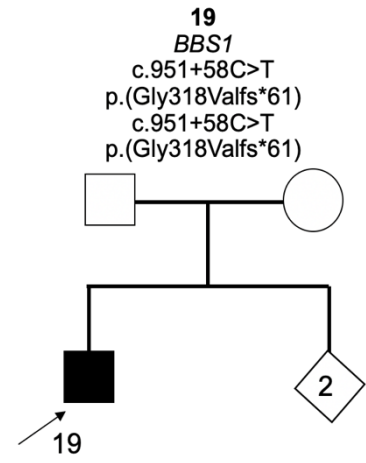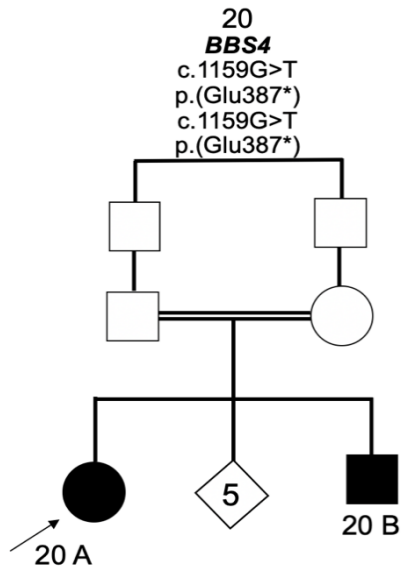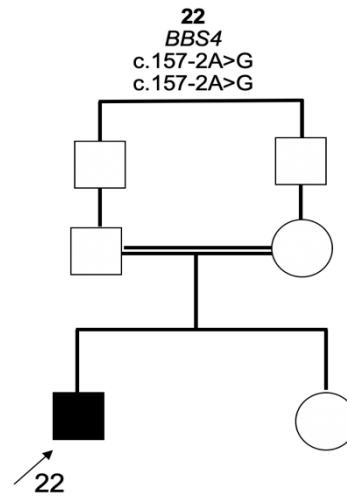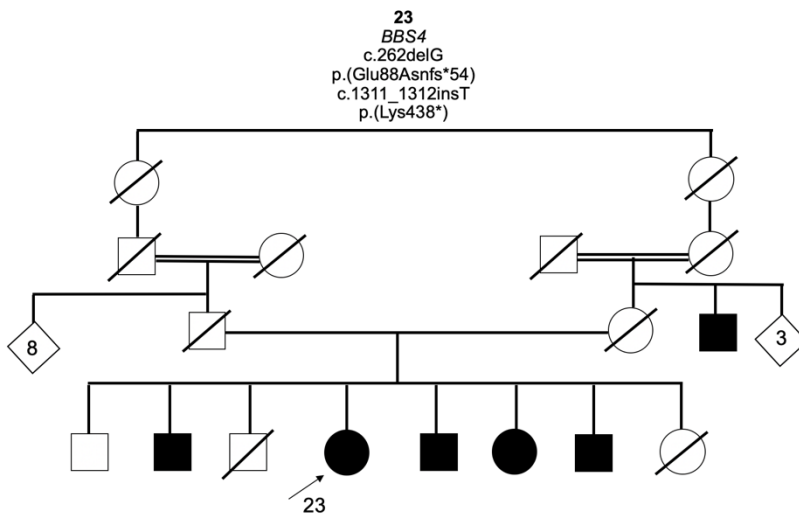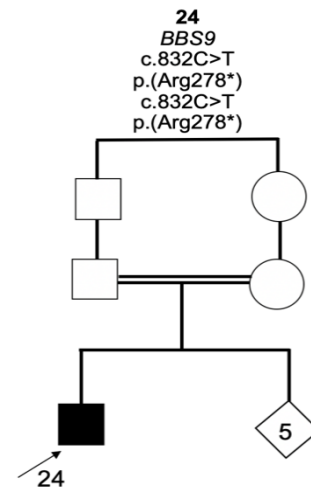

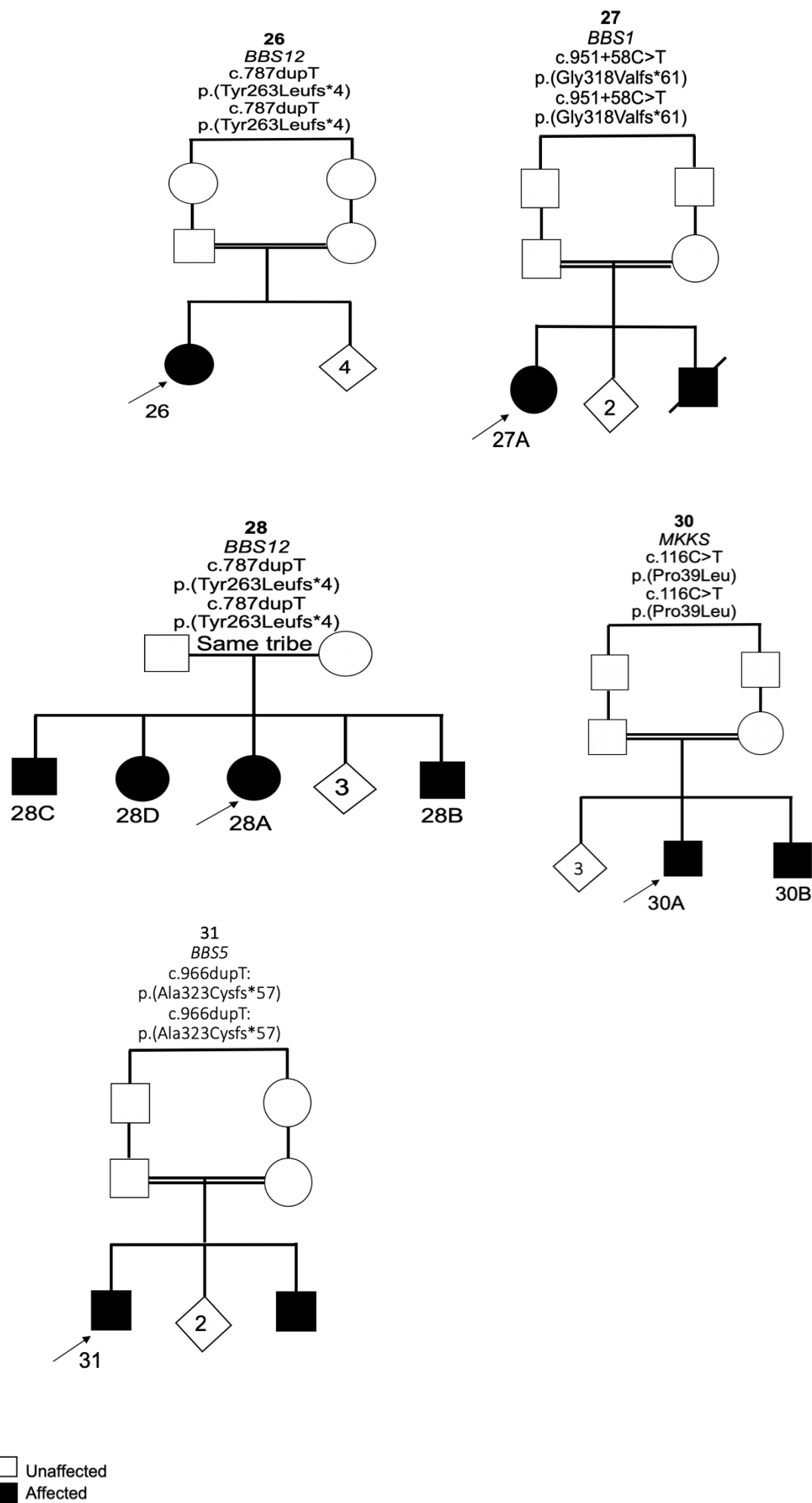

**Figure S6:** Pedigrees of 23 of the 31 probands; pedigrees of nine probands were not available: 1, 4, 6, 13, 17, 21, 25, 27 and 29.

Supplement: Supplementary file 1 [file genes-15-00762-s001.zip › Figure S6 pedigree of probands.pdf]
